# Prolactin-Releasing Hormone Receptor (PRLHR) enhances radiosensitivity and exacerbates DNA damage in glioblastoma post-irradiation by inhibiting Y-box-binding protein-1 (YBX1) nuclear translocation: a novel perspective on precision radiotherapy

**DOI:** 10.1186/s43556-026-00503-7

**Published:** 2026-06-29

**Authors:** Yuning Qiu, Jing Zhang, Jingdian Liu, Zilong Wang, Zeyu Ma, Minkai Wang, Qimeng Wang, Xianzhi Liu, Dongming Yan, Zhenyu Zhang

**Affiliations:** 1 Department of Surgery ICU, The First Affiliated Hospital of Zhengzhou University, Zhengzhou, Henan China; 2 Department of Neurosurgery, The First Affiliated Hospital of Zhengzhou University, Zhengzhou, Henan China; 3 Department of Pathology, Henan Provincial Chest Hospital, Zhengzhou, Henan China; 4 Department of Pathology, The First Affiliated Hospital of Zhengzhou University, Zhengzhou, Henan China

**Keywords:** Glioblastoma, Radiosensitivity, DNA damage, YBX1, Nuclear translocation

## Abstract

Radiotherapy is a cornerstone for glioblastoma multiforme (GBM), yet its efficacy is severely hampered by intrinsic radioresistance, the molecular basis of which remains elusive, and overcoming this resistance poses a persistent challenge for GBM therapeutics. This study aims to identify novel key regulators of radiosensitivity in glioblastoma (GBM), with the goal of developing potent radiosensitizers. By analyzing patient cohorts, we identified prolactin-releasing hormone receptor (PRLHR) as a differentially expressed molecule, with significantly elevated levels in glioma specimens from the long PFS group compared to the short PFS group. This discovery prompted us to investigate PRLHR’s role in modulating GBM radiosensitivity and DNA repair. Using in vitro and in vivo models, we found PRLHR overexpression enhanced GBM radiosensitivity by promoting DNA damage accumulation and attenuating repair pathways. Mechanistically, PRLHR interacts with Y-box-binding protein-1 (YBX1), inhibiting its nuclear translocation to reduce nuclear YBX1 levels, thereby compromising DNA repair efficiency. We further demonstrated repressor element-1 binding transcription factor (REST) directly binds the *PRLHR* promoter to transcriptionally repress its expression. Notably, degradation of REST by the antagonist X5050 upregulated PRLHR, increased DNA damage, and enhanced the therapeutic efficacy of radiotherapy in preclinical models. Our findings delineate PRLHR as a critical regulator of GBM radiosensitivity and establish X5050 as a promising radiosensitizing agent, offering a novel precision strategy to overcome radioresistance.

**Supplementary Information:**

The online version contains supplementary material available at 10.1186/s43556-026-00503-7.

## Introduction

Glioblastoma (GBM) is the most common and aggressive primary malignant brain tumor in adults, representing approximately 50.1% of all malignant tumors of the central nervous system [1]. Based on the fifth edition of the World Health Organization (WHO) Classification of Central Nervous System Tumors (2021), adult diffuse gliomas are categorized into three major groups: (1) astrocytoma, isocitrate dehydrogenase(IDH)-mutant; (2) oligodendroglioma, IDH-mutant and 1p/19q-codeleted; and (3) GBM, IDH-wildtype [2]. Despite contemporary multimodal therapies—including maximal safe resection, radiotherapy, and temozolomide chemotherapy—the prognosis remains dismal [3]. Recent clinical evaluations in 2024 and 2025 indicate that the median overall survival persists at approximately 15 months, with a 5-year survival rate below 7%, emphasizing the urgent need for therapeutic innovation [3, 4].

Radiotherapy, a standard treatment for GBM, kills tumor cells by directly or indirectly inducing DNA double-strand breaks (DSBs) and single-strand breaks (SSBs) [5, 6]. However, GBM’s inherent heterogeneity fosters radioresistance, severely compromising radiotherapy efficacy [7]. While radiation-induced DNA damage drives tumor cell death, DNA damage and repair are governed by intricate intracellular and extracellular mechanisms [8]. Tumor cells can acquire radioresistance via intrinsic factors or tumor microenvironment (TME) influences, causing treatment failure and recurrence [9]. For example, cells are highly radiosensitive under physoxia (pO₂ ≈ 40 mmHg): 1 Gy X-rays induce ~3000 base lesions, 1000 SSBs, and 40 DSBs [10]. The “oxygen fixation hypothesis” posits that under hypoxic conditions, DNA radicals formed by ionization can be chemically restored to their original form through hydrogen atom transfer from sulfhydryl groups (e.g., glutathione), a process known as chemical restitution, thereby conferring radioresistance [11]. Additionally, radiosensitivity correlates with DNA repair capacity, TME, cell cycle arrest, cancer stem cells, reactive oxygen species (ROS) levels, and tumor metabolism [12–19]. Despite progress in radiation oncology, radioresistance remains a key barrier in GBM treatment. Thus, clarifying its molecular mechanisms and developing targeted radiosensitization strategies are vital to enhance GBM patient outcomes.

Our pilot study identified common differentially expressed genes (DEGs) shared across different datasets by comparing radiotherapy-treated glioma patients with long versus short PFS/OS. Through this intersection analysis, PRLHR emerged as a potential candidate. Prolactin-releasing hormone receptor (PRLHR), also known as G protein-coupled receptor 10, serves as the receptor for prolactin-releasing peptide (PrRP) [20]. Under normal physiological conditions, it is primarily expressed in the hypothalamus. PRLHR not only mediates physiological responses to centrally administered PrRP but also plays roles in other processes such as feeding behavior, energy expenditure, obesity, and blood pressure regulation [21]. Relevant studies have indicated that PRLHR is highly expressed in uterine fibroids and is associated with tumor cell proliferation [20]. Concurrently, studies have demonstrated that PRLHR expression is negatively correlated with immune cell infiltration [22]. Liu et al. suggested that PRLHR may function as a tumor suppressor that inhibits the progression of low-grade gliomas [23]. Furthermore, in GBM, *PRLHR* mRNA is reportedly downregulated [24]. However, the specific roles and molecular mechanisms of PRLHR in GBM, particularly its impact on radiotherapy, remain uncharacterized. Whether PRLHR regulates radiosensitivity in GBM remains elusive.

Based on the inconsistent role of PRLHR in different tumors and the lack of research on its role in GBM, combined with the clinical demand for improving GBM radiosensitivity, we aimed to explore the association between PRLHR and GBM radiosensitivity and its molecular mechanism, thereby providing a new target for GBM radiosensitization treatment.

## Results

### PRLHR is potentially linked to glioma radiosensitivity and clinical outcomes

**Fig. 1:**
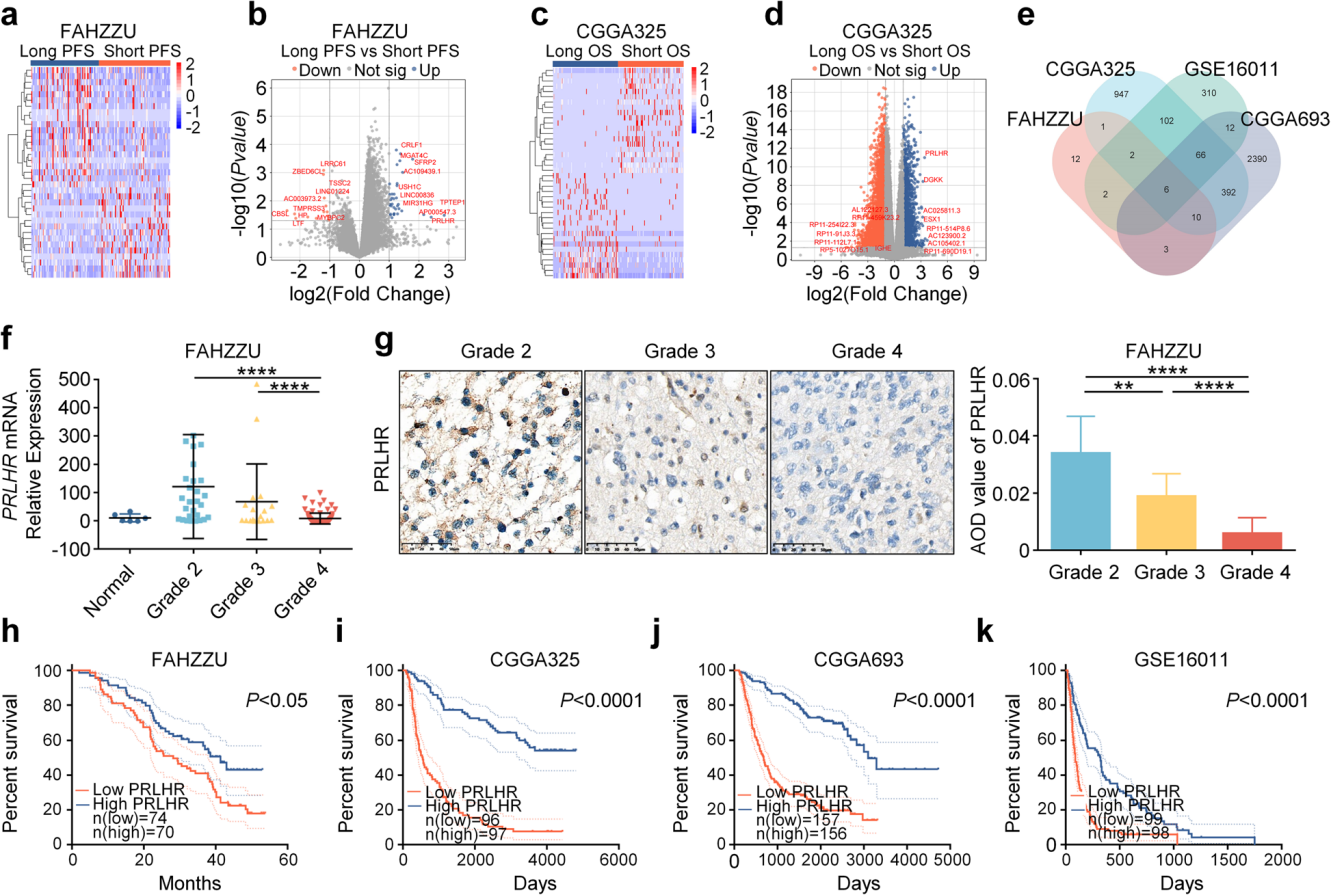
PRLHR is potentially linked to glioma radiosensitivity and clinical outcomes. **a** Heatmap showing differentially expressed genes (DEGs) between long PFS and short PFS glioma groups, based on RNA sequencing data from the First Affiliated Hospital of Zhengzhou University (FAHZZU). **b** Volcano plot illustrating gene expression differences between long PFS and short PFS glioma groups in the FAHZZU dataset. **c** Heatmap displaying differential gene expression between long OS and short OS glioma groups based on the CGGA325 dataset. **d** Volcano plot depicting gene expression differences between long OS and long OS glioma groups in the CGGA325 dataset. **e** Venn diagram showing the intersection of upregulated genes in long PFS/OS groups across the FAHZZU, CGGA325, CGGA693, and GSE16011 datasets. **f** Comparison of *PRLHR* mRNA expression levels among radiotherapy-treated glioma patients of different WHO grades (normal brain, *n* = 6; grade 2, *n* = 28; grade 3, *n* = 18; grade 4, *n* = 98), based on FAHZZU sequencing data. **g** Immunohistochemistry (IHC) detection of PRLHR expression in glioma tissues of different WHO grades from FAHZZU (including 21 cases of WHO grade 2, 10 cases of WHO grade 3, and 30 cases of WHO grade 4). Left panel: Representative IHC images. Right panel: Quantitative analysis of PRLHR expression levels. Scale bars, 50 μm. AOD, average optical density. **h**–**k** Kaplan–Meier curves for overall survival (OS) of glioma patients treated with radiotherapy, stratified by high *PRLHR* expression (above the median) and low *PRLHR* expression (below the median) in the FAHZZU (**h**), CGGA325 (**i**), CGGA693 (**j**), and GSE16011 (**k**) datasets. *p < 0.05, **p < 0.01, ***p < 0.001, and ****p < 0.0001

To identify the key molecules that potentially regulate radiosensitivity in gliomas, we enrolled 144 adult patients with diffuse gliomas who received postoperative radiotherapy. We obtained progression-free survival (PFS) and overall survival (OS) data through follow-up and performed RNA-seq on postoperative tumor specimens. The median PFS of 144 patients was approximately 19 months; therefore, we selected 19 months as the cut-off value. Based on this cut-off value, patients were stratified into the long PFS group (PFS > cut-off) and the short PFS group (PFS ≤ cut-off). RNA-seq analysis revealed significant differential expression of multiple genes between the two groups. These genes included *TPTEP1* and *SFRP2*, which have been previously documented to be associated with radiosensitivity in glioma [25, 26]. Notably, PRLHR expression was significantly elevated in the long PFS group relative to the short PFS group, ranking as the second most upregulated gene (Fig. 1a–b). Furthermore, we obtained data from the CGGA and GEO databases and selected glioma patients who had undergone radiotherapy. These patients were similarly stratified into long and short OS groups to screen for differentially expressed genes between these two cohorts. Consistent with our gene expression results, we observed that in the CGGA325 dataset, *PRLHR* was significantly upregulated in the long OS group, ranking as the third most upregulated gene (Fig. 1c–d). We then identified genes that were significantly upregulated (fold change > 2) in the long PFS/OS group across the different datasets and took their intersection. This analysis revealed a total of 6 overlapping genes, including *PRLHR, SPHKAP, CACNG2, ABCC8, USH1C, MGAT4C* (Fig. 1e). Besides *PRLHR*, the other five genes have been previously reported to exert their roles in glioma or be associated with tumor radiosensitivity or DNA repair [27–33]. These results highlight PRLHR as a molecule of interest, implying its possible dual role in modulating glioma radiosensitivity and serving as a prognostic indicator for patients receiving radiotherapy.

Subsequently, we investigated the expression of *PRLHR* mRNA and protein in glioma tissues. The results showed that in glioma, the level of *PRLHR* mRNA decreases with increasing WHO grade, and exhibits the lowest expression level in grade 4 GBM (Fig. 1f). IHC confirmed that PRLHR protein levels were inversely correlated with the WHO grade (Fig. 1g). Additionally, we stratified patients into *PRLHR* high-expression and low-expression groups based on the median of *PRLHR* mRNA expression levels, comparative analysis of overall survival (OS) between high and low *PRLHR* expression groups across multiple datasets revealed that high *PRLHR* expression was associated with improved survival outcomes in patients following radiotherapy (Fig. 1h–k). A Cox proportional hazards regression model was performed on the FAHZZU dataset, incorporating age, sex, *PRLHR* expression level, WHO grade, complete resection, 1p/19q codeletion status, and chemotherapy treatment was performed. As expected, the analysis confirmed that complete resection (hazard ratio = 0.597) and chemotherapy (hazard ratio = 0.086) were significantly associated with better clinical outcomes, whereas WHO grade 4 (hazard ratio = 3.748) was a significant predictor of poor survival. Importantly, even after adjusting for these well-established clinical factors, high PRLHR expression remained independently associated with favorable survival outcomes in glioma patients undergoing radiotherapy (hazard ratio = 0.609) (Fig. S1a). Consistent findings were observed across the CGGA325, CGGA693, and GSE16011 cohorts when analyzed using the Cox proportional hazards regression model (Fig. S1b–d). These findings suggest that glioma patients with high PRLHR expression have improved survival outcomes following radiotherapy, indicating that PRLHR may play an important role in regulating the radiosensitivity of gliomas (including GBM).

### *PRLHR* overexpression enhances radiosensitivity in GBM cells

**Fig. 2:**
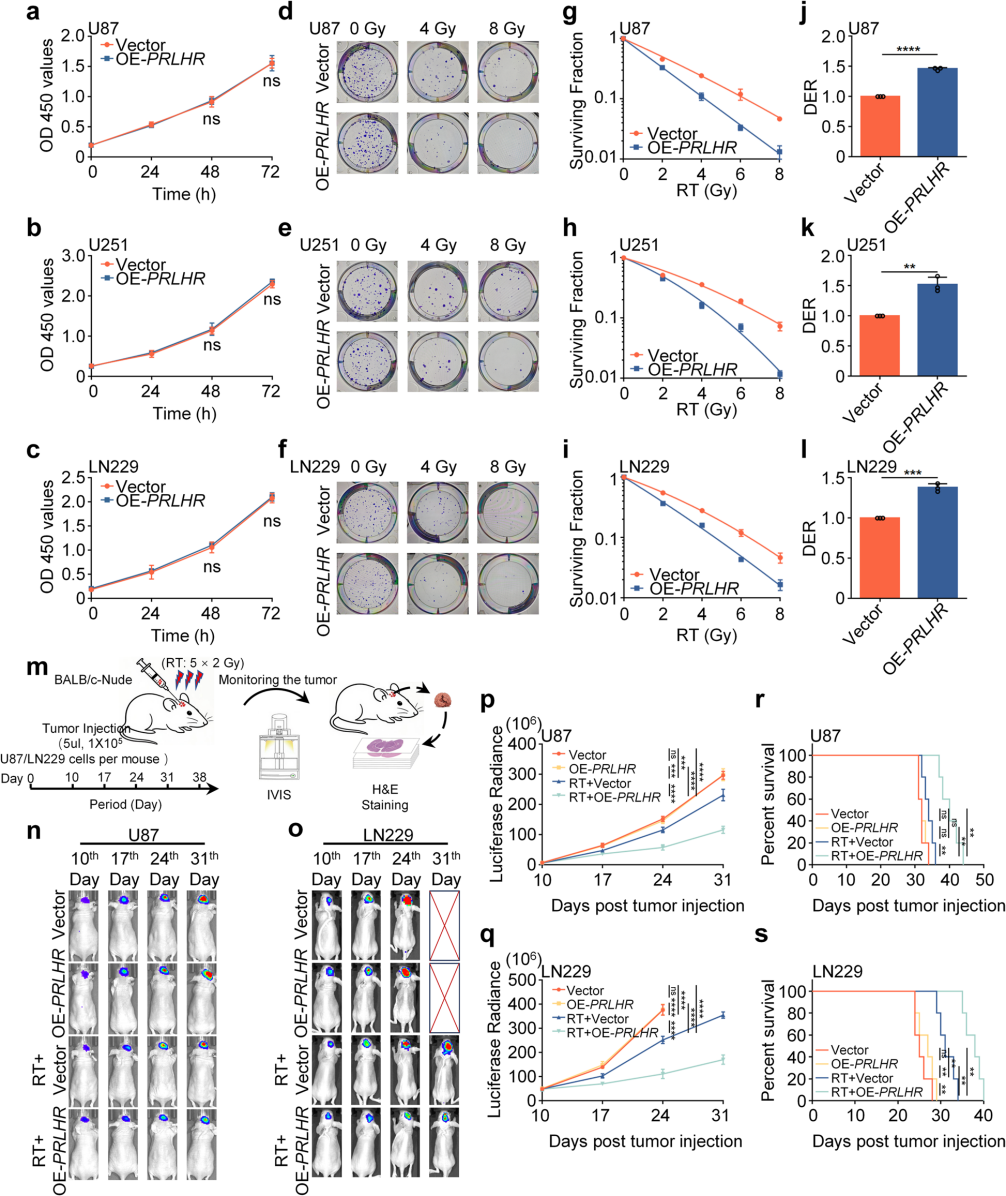
*PRLHR* overexpression enhances radiosensitivity in GBM cells. **a**-**c** Cell viability assays of U87 (**a**), U251 (**b**) and LN229 (**c**) cells (*PRLHR*-overexpressing and control) for evaluating proliferation rates. **d**-**f** Representative images from post-irradiation colony formation assays of U87 (**d**), U251(**e**) and LN229 (**f**) cells overexpressing *PRLHR* and their control cells (*n* = 3). **g**-**i** Surviving fractions of U87 (**g**), U251 (**h**) and LN229 (**i**) cells exposed to different doses of irradiation, with the survival curves fitted using the linear-quadratic (LQ) model. **j**-**l** Dose enhancement ratio (DER) for U87 (**j**), U251 (**k**) and LN229 (**l**) cells overexpressing *PRLHR*. **m** Schematic illustration of the orthotopic GBM model established in BALB/c nude mice. U87/LN229 cells (1 × 10^5^) were implanted intracranially. Ten days post-implantation, mice were randomized into four groups: Vector, OE-*PRLHR*, RT + Vector, and RT + OE-*PRLHR* (*n* = 5 per group). Tumor burden was monitored via bioluminescence imaging, followed by fractionated radiotherapy (5 × 2 Gy). Upon study termination, mice were euthanized, and tumor tissues were excised for hematoxylin and eosin (H&E) staining. **n**, **o** Representative in vivo bioluminescence images (showing one representative mouse per group) monitoring intracranial tumor growth in U87 (**n**) and LN229 (**o**) glioblastoma models (*n* = 5 per group). **p**, **q** Quantification of tumor bioluminescence radiance over time in U87 (**p**) and LN229 (**q**) tumor-bearing mice. **r**, **s** Kaplan–Meier survival analysis of the indicated groups in U87 (**r**) and LN229 (**s**) intracranial tumor models. *p < 0.05, **p < 0.01, ***p < 0.001, and ****p < 0.0001

Subsequently, we focused on GBM to further elucidate the role of PRLHR in this malignancy. We established *PRLHR*-overexpressing GBM cell lines (U87, U251, and LN229) and confirmed overexpression efficiency using qPCR and western blotting (Fig. S2a–f). Cell viability assays showed that overexpression of *PRLHR* had no significant effect on the proliferative capacity of U87, U251 and LN229 cells (Fig. 2a–c). Interestingly, post-irradiation colony formation assays demonstrated that *PRLHR* overexpression decreased the Surviving Fraction (SF) of U87, U251 and LN229 cells following irradiation, with a dose enhancement ratio (DER) > 1, indicating that *PRLHR* overexpression enhances the radiosensitivity of GBM cells (Fig. 2d–l). Furthermore, we established orthotopic intracranial GBM mouse models using both U87 and LN229 cells to validate these findings in vivo. Tumors in the radiotherapy (RT) group were subjected to fractionated irradiation (5 × 2 Gy; 2 Gy/day for 5 consecutive days) (Fig. 2m). Consistent with our in vitro observations, in vivo assays demonstrated that *PRLHR* overexpression alone did not significantly alter GBM tumor progression compared to the control group. However, in the presence of RT, *PRLHR*-overexpressing tumors exhibited a superior therapeutic response compared to controls. Specifically, tumor growth was markedly suppressed, and overall survival was significantly prolonged in the *PRLHR*-overexpression group (Fig. 2n–s). Notably, no significant differences in body weight were observed across the groups (Fig. S2g–h). Collectively, these results suggest that PRLHR modulates the radiosensitivity of GBM, and its overexpression sensitizes GBM cells to radiotherapy.

### PRLHR inhibits DNA double-strand break repair in GBM cells following irradiation

**Fig. 3:**
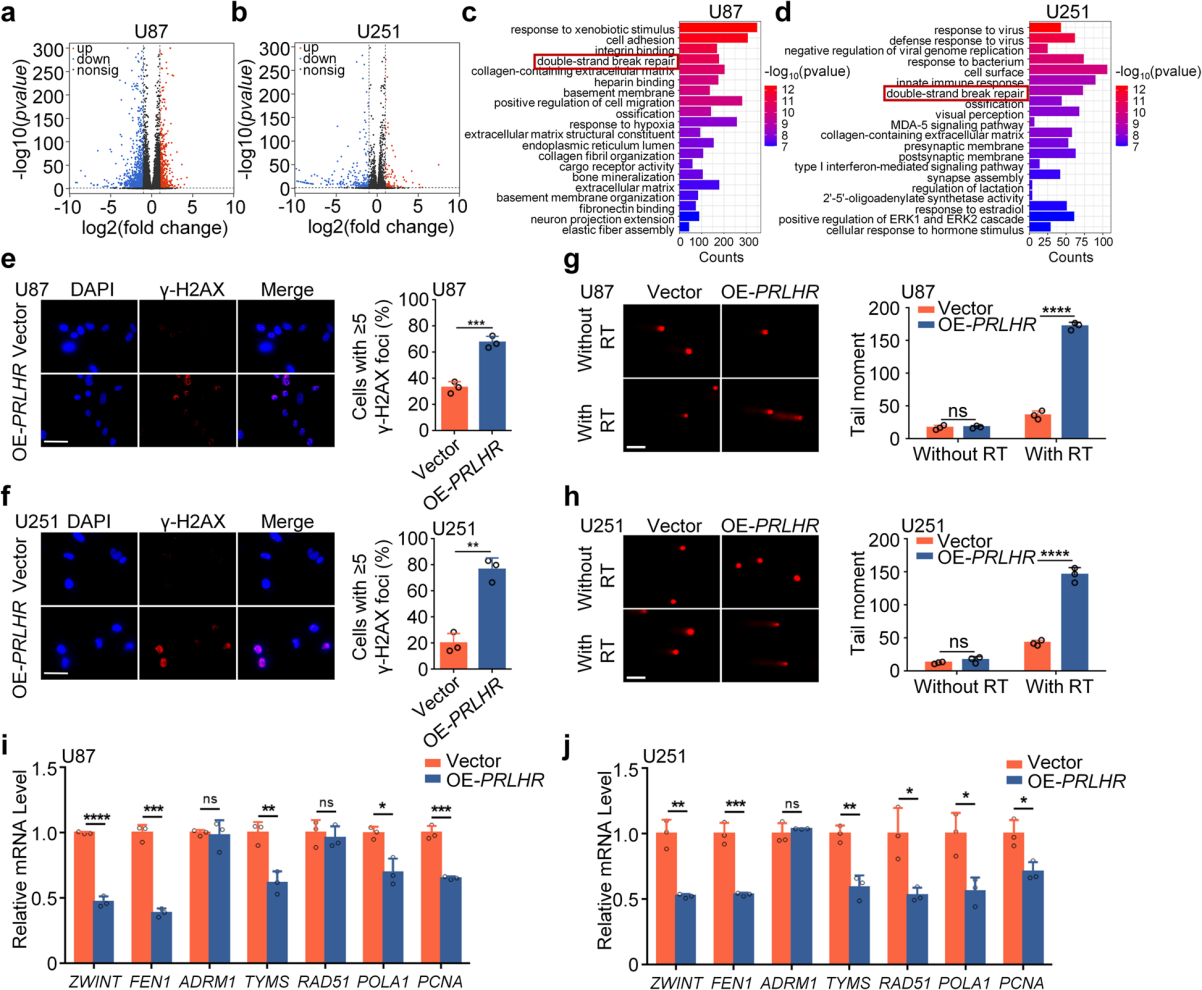
PRLHR inhibits DNA double-strand break repair in GBM cells following irradiation. **a**, **b** Volcano plots illustrating differential gene expression between *PRLHR*-overexpressing U87 cells (**a**) and U251 cells (**b**) compared to their respective control cells. **c**, **d** Gene Ontology (GO) enrichment analysis of *PRLHR*-overexpressing U87 cells (**c**) and U251 cells (**d**). **e**, **f** Detection of DNA DSBs assessed by γ-H2AX levels in U87 cells (**e**) and U251 cells (**f**) at 24 h post-irradiation (4 Gy) (*n* = 3). Left panels: Representative immunofluorescence images of γ-H2AX foci; Right panels: Quantification of γ-H2AX foci per cell. Scale bars, 50 μm. **g**, **h** total DNA damage in irradiated (4 Gy, 24 h post-irradiation) and non-irradiated U87 (**g**) and U251 (**h**) cells detected by comet assay (*n* = 3). Left panels: Representative fluorescence images of comet assays; Right panels: Quantification of tail moment. Scale bars, 50 μm. **i**, **j** qPCR analysis of expression changes in DNA repair-related genes in *PRLHR*-overexpressing U87 cells (**i**) and U251 cells (**j**). *p < 0.05, **p < 0.01, ***p < 0.001, and ****p < 0.0001 by Student’s t-test

Radiation sensitivity in tumors is influenced by factors such as DNA repair, the TME, cell cycle, ROS distribution, and tumor metabolism [12–19]. To further investigate the mechanism by which PRLHR regulates radiation sensitivity in GBM, we performed RNA-seq of *PRLHR*-overexpressing U87 and U251 cell lines and their corresponding controls. The results revealed that *PRLHR* overexpression alters the expression of hundreds of genes. Following Gene Ontology enrichment analysis of the sequencing data, significant enrichment of double-strand break repair pathways was observed in both U87 and U251 cells (Fig. 3a–d).

To confirm these findings, the three GBM cell lines (U87, U251, and LN229) were irradiated at a dose of 4 Gy. To evaluate DNA damage at 24 h post-irradiation, we employed γ-H2AX immunofluorescence staining to detect DSBs and the alkaline comet assay to assess total DNA strand breaks. Immunofluorescence analysis demonstrated that γ-H2AX foci were significantly increased in *PRLHR*-overexpressing U87, U251, and LN229 cells compared to controls after irradiation (Fig. 3e–f and Fig. S3a). The comet assay revealed that these cells exhibited longer comet tails following irradiation than control cells did, whereas no difference in tail length was observed between the two groups without irradiation (Fig. 3g–h and Fig. S3b). These results indicate that *PRLHR* overexpression increases the levels of DNA damage in GBM cells after irradiation. Previous studies have shown that activation of the G2/M checkpoint pathway during DNA damage prevents premature entry into mitosis (M phase), allowing time for DNA repair [34]. We examined the expression of key genes involved in DNA repair and the G2/M checkpoint pathway and found that *PRLHR* overexpression downregulated the expression of *ZWINT, FEN1, TYMS, POLA1*, and *PCNA* across all three cell lines compared to their respective controls (Fig. 3i–j and Fig. S3c).

### PRLHR binds to YBX1 and inhibits its nuclear translocation, thereby increasing DNA damage

**Fig. 4:**
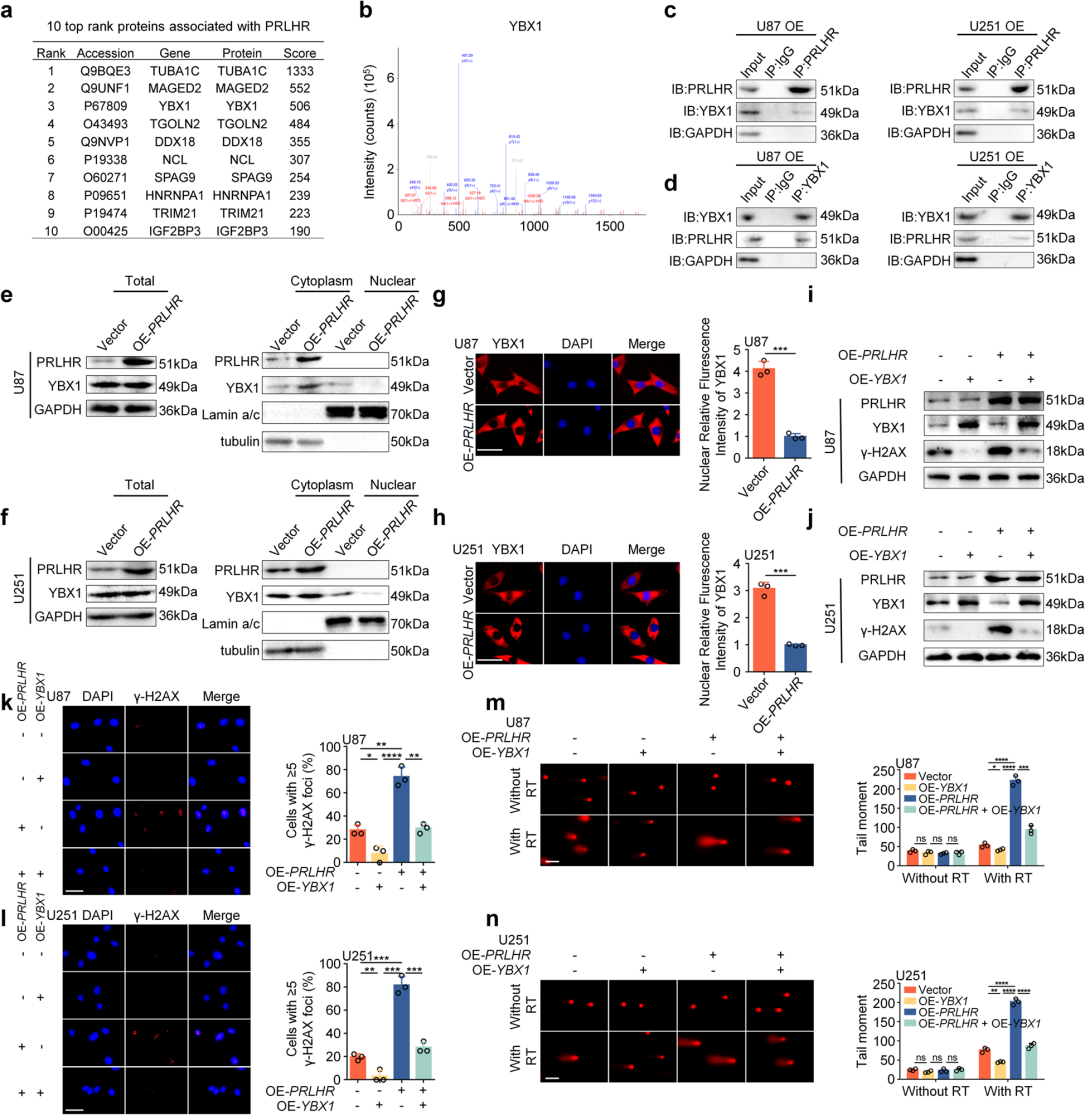
PRLHR binds to YBX1 and inhibits its nuclear translocation, thereby increasing DNA damage. **a** The top 10 proteins potentially associated with PRLHR were isolated by immunoprecipitation and identified by mass spectrometry in *PRLHR*-overexpressing U87 (U87 OE) cells. **b** Mass spectrum of YBX1. **c**, **d** Western blot analysis of PRLHR and YBX1 proteins using co-immunoprecipitation with anti-PRLHR (**c**) or anti-YBX1 (**d**) antibodies in U87 OE and U251 OE cells. Left panels: U87 OE cells; Right panels: U251 OE cells. **e**, **f** Western blot detection of PRLHR and YBX1 expression levels in whole-cell lysates, cytoplasmic fractions, and nuclear fractions of U87 cells (**e**) and U251 cells (**f**). **g**, **h** Immunofluorescence analysis of the subcellular localization of YBX1 in U87 cells (**g**) and U251 cells (**h**). Left panels: Representative images of YBX1 subcellular localization (*n* = 3). Scale bar, 50 μm. Right panels: Quantification of the relative nuclear fluorescence intensity of YBX1. **i**, **j** Western blot analysis of γ-H2AX expression levels in U87 cells (**i**) and U251 cells (**j**) with *PRLHR* and/or *YBX1* overexpression following 4 Gy irradiation for 24 h. **k**, **l** Immunofluorescence detection of γ-H2AX foci in *PRLHR* and/or *YBX1* overexpressing U87 cells (**k**) and U251 cells (**l**) at 24 h post-irradiation (4 Gy) (*n* = 3). Left panels show representative γ-H2AX foci; Right panels show quantification data. Scale bars, 50 μm. **m**, **n** Comet assay was performed to detect total DNA damage in irradiated (4 Gy, 24 h post-irradiation) and non-irradiated U87 cells (**m**) and U251 cells (**n**) with overexpression of *PRLHR* and/or *YBX1* (*n* = 3). Left panels show representative comet assay fluorescence images; Right panels show quantification data. Scale bars, 50 μm. *p < 0.05, **p < 0.01, ***p < 0.001, and ****p < 0.0001

To further investigate the mechanism by which PRLHR suppresses DNA repair, we performed immunoprecipitation using lysates from *PRLHR*-overexpressing U87 cells, followed by mass spectrometry (MS) to identify potential interacting proteins. The results revealed that at least 158 proteins potentially interact with PRLHR (Table S3). Among these, YBX1 drew particular interest, as studies have shown that in colon cancer, YBX1 binds to the *BRCA1* promoter, increases BRCA1 expression, thereby enhancing DNA repair [35]. Protein abundance-based scoring identified YBX1 (ranked third) as a strong candidate, and MS spectra confirmed its presence in the immunoprecipitates (Fig. 4a–b). Subsequently, co-IP assays confirmed a direct interaction between PRLHR and YBX1 in U87, U251, and LN229 cells (Fig. 4c–d and Fig. S4a–b).

Subcellular localization of YBX1 is critically linked to DNA repair. Its nuclear localization protects DNA from damage, whereas its cytoplasmic localization does not [35]. Therefore, we examined the effect of PRLHR on the subcellular localization of YBX1. Western blot analysis showed that *PRLHR* overexpression in U87, U251, and LN229 cells decreased nuclear YBX1 localization while increasing its cytoplasmic localization, without affecting total YBX1 levels (Fig. 4e–f and Fig. S4c). Immunofluorescence assays also demonstrated reduced nuclear expression of YBX1 in *PRLHR*-overexpressing cells across all three cell lines compared to their respective controls (Fig. 4g–h and Fig. S4d). These findings suggest that the binding of PRLHR to YBX1 may inhibit the nuclear import of YBX1, thereby reducing its nuclear accumulation.

To further confirm whether PRLHR increases DNA damage via YBX1, we performed co-overexpression of *PRLHR* and *YBX1* in U87, U251, and LN229 cell lines and assessed the resultant effects on DNA damage. Western blot results showed that *YBX1* overexpression reduced γ-H2AX levels following irradiation. Furthermore, *YBX1* overexpression reversed the increase in post-irradiation γ-H2AX levels induced by *PRLHR* overexpression (Fig. 4i–j and Fig. S4e). Consistently, immunofluorescence (Fig. 4k–l and Fig. S4f) and comet assay (Fig. 4m–n and Fig. S4g) demonstrated that *YBX1* overexpression decreased γ-H2AX foci and comet tail moments in these cell lines post-irradiation, while also abrogating the *PRLHR* overexpression-mediated elevation of these DNA damage markers under irradiation. Notably, *YBX1* overexpression exerted no significant effect on γ-H2AX foci or comet tail moments in the absence of irradiation. Collectively, these results indicate that PRLHR interacts with YBX1, inhibits its nuclear import, diminishes YBX1 nuclear localization, and thereby enhances DNA damage in GBM cells post-irradiation.

### REST binds to the *PRLHR* promoter and negatively regulates PRLHR expression

**Fig. 5:**
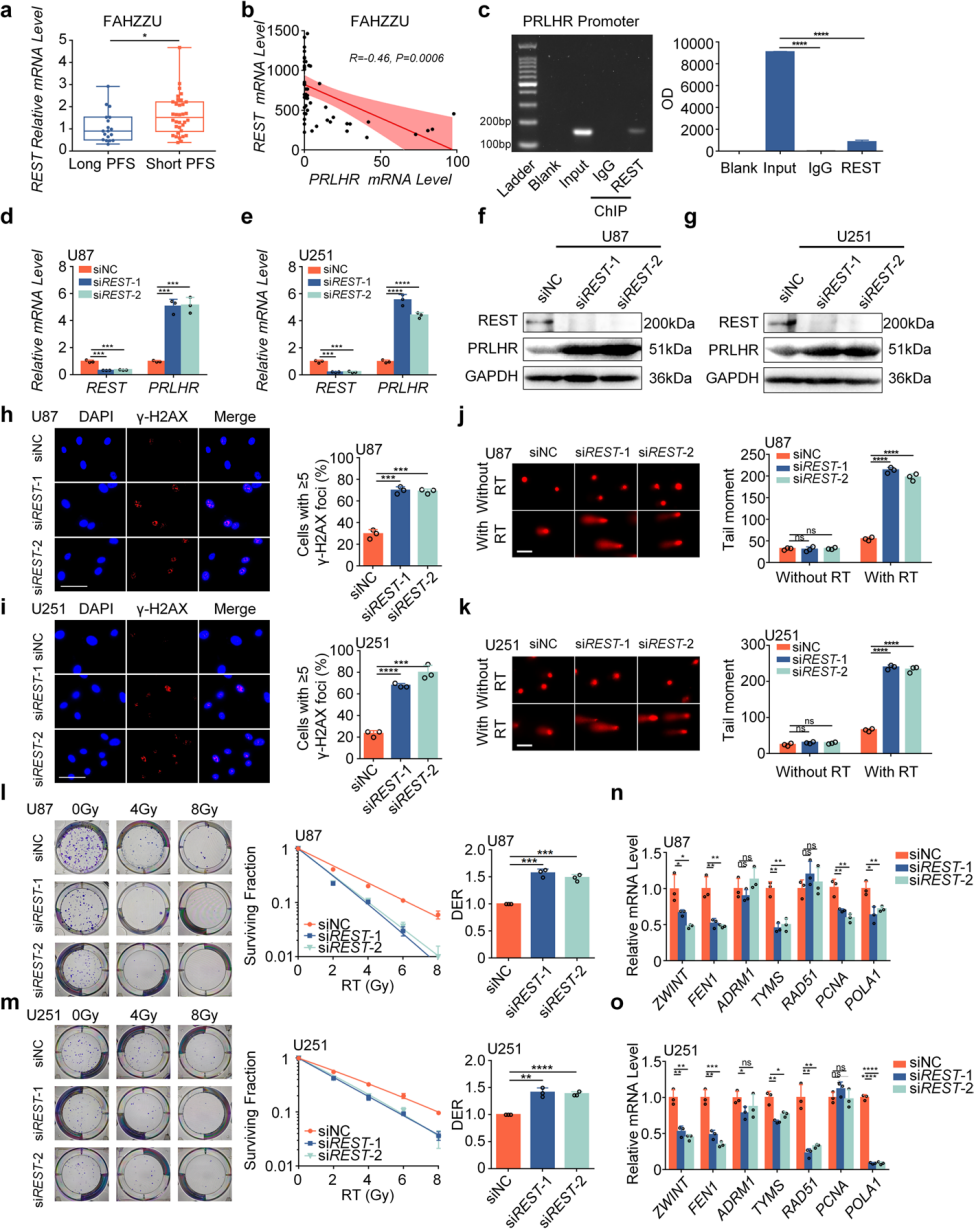
REST binds to the *PRLHR* promoter and negatively regulates PRLHR expression. **a** Comparison of *REST* mRNA expression levels between long PFS and short PFS groups in GBM patients (collected from FAHZZU, First Affiliated Hospital of Zhengzhou University). **b** Correlation analysis between *PRLHR* mRNA and *REST* mRNA expression in tumor tissues (GBM patients from FAHZZU, First Affiliated Hospital of Zhengzhou University). **c** Chromatin immunoprecipitation (ChIP) assay detecting the binding of REST to the PRLHR promoter (*n* = 3). **d**, **e** qPCR analysis of *PRLHR* mRNA expression following *REST* knockdown in U87 cells (**d**) and U251 cells (**e**) (*n* = 3). **f**, **g** Western blot detection of PRLHR protein levels after *REST* knockdown in U87 cells (**f**) and U251 cells (**g**). **h**, **i** Immunofluorescence analysis of γ-H2AX foci in *REST*-knockdown U87 cells (**h**) and U251 cells (**i**) at 24 h post-irradiation (4 Gy) (*n* = 3). Left panels: representative γ-H2AX foci; Right panels: quantitative data. Scale bars, 50 μm. **j**, **k** Comet assay was performed to detect total DNA damage in irradiated (4 Gy, 24 h post-irradiation) and non-irradiated *REST* knockdown U87 cells (**j**) and U251 cells (**k**) (*n* = 3). Left panels: representative comet assay images; Right panels: quantification data. Scale bars, 50 μm. **l**, **m** Colony formation assay analyzing cell survival and dose enhancement ratio (DER) in *REST*-knockdown U87 cells (**l**) and U251 cells (**m**) (*n* = 3). Left panels: representative colony images; Middle panels: survival fractions at different radiation doses, with curves fitted using the linear-quadratic (LQ) model; Right panels: Dose Enhancement Ratio (DER). **n**, **o** qPCR analysis of DNA repair-related gene expression changes in *REST*-knockdown U87 cells (**n**) and U251 cells (**o**) (*n* = 3). *p < 0.05, **p < 0.01, ***p < 0.001, and ****p < 0.0001 by Student’s t-test

Varghese et al. reported that loss of repressor element-1 binding transcription factor (REST) increases PRLHR expression in uterine fibroids [20]. To investigate this mechanism in GBM, we first compared *REST* mRNA levels between long and short PFS cohorts. Notably, REST expression was significantly lower in the long PFS group compared to the short PFS group (Fig. 5a). Correlation analysis revealed a negative correlation between *REST* and *PRLHR* mRNA expression in GBM (Fig. 5b). To further investigate the mechanism by which REST regulates PRLHR expression, we performed ChIP-PCR assays to assess REST binding to the *PRLHR* promoter in U87 cells. The results demonstrated that REST binds to the *PRLHR* promoter (Fig. 5c), which may underlie its negative regulation of PRLHR.

To further validate the repressive role of REST on PRLHR expression and its impact on GBM radiosensitivity, we used siRNA to knock down REST expression in U87, U251, and LN229 cells. Both qPCR and western blotting results showed that *REST* knockdown increased *PRLHR* mRNA and protein levels across all three cell lines (Fig. 5d–g and Fig. S5a–b). Concurrently, immunofluorescence assays confirmed that *REST* knockdown increased the formation of γ-H2AX foci following irradiation (Fig. 5h–i and Fig. S5c). Comet assays similarly demonstrated that REST knockdown increased comet tail moments in GBM cells post-irradiation (Fig. 5j–k and Fig. S5d). Colony formation assays further showed that REST knockdown reduced the surviving fraction and increased the radiosensitivity of U87, U251, and LN229 cells (Fig. 5l–m and Fig. S5e). Furthermore, compared with the control group, REST inhibition downregulated the mRNA expression of key genes in DNA repair-related pathways, such as *ZWINT, FEN1, TYMS*, and *POLA1*, in these cells (Fig. 5n–o and Fig. S5f).

**Fig. 6:**
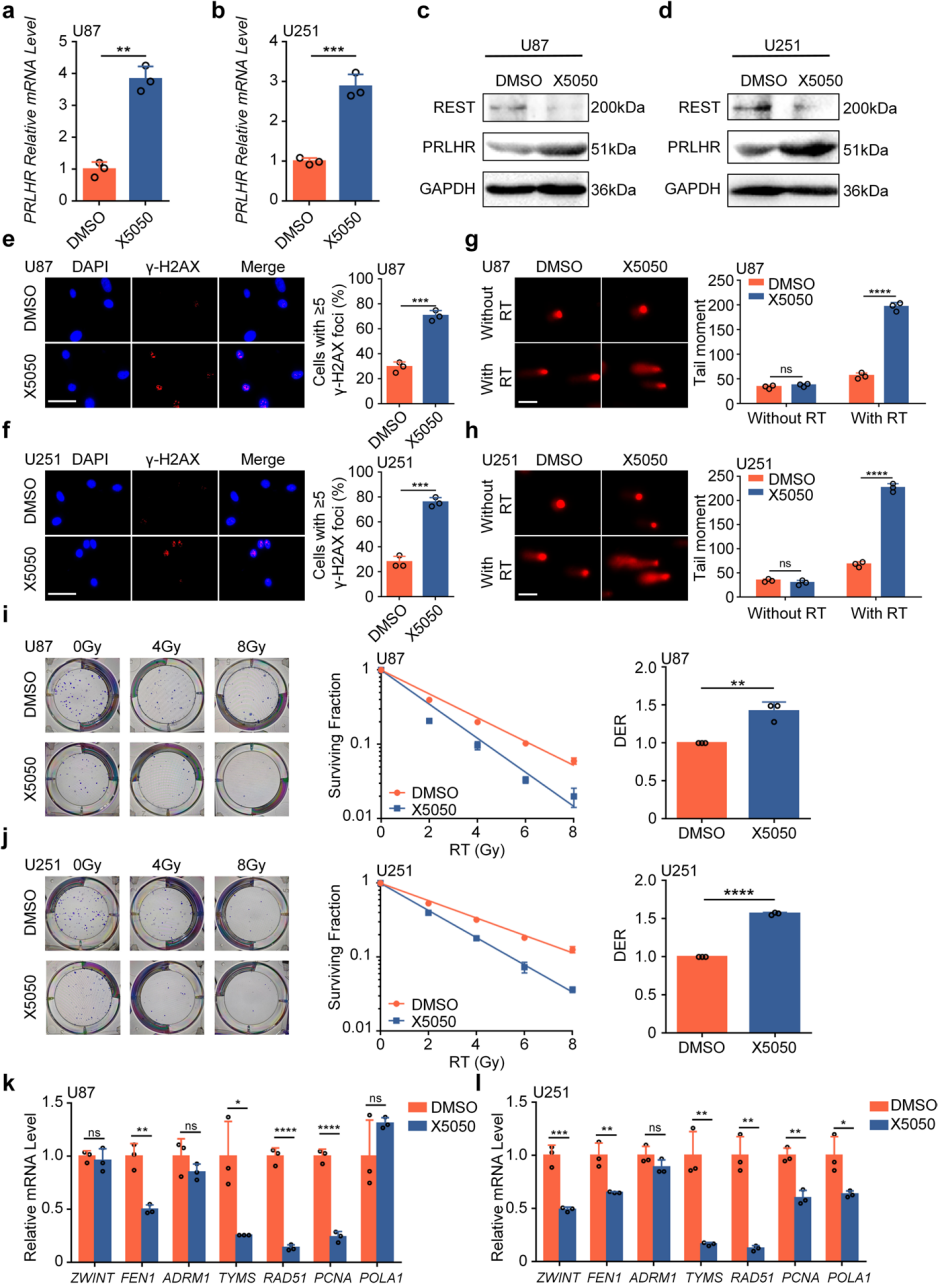
REST inhibitor X5050 enhances GBM radiosensitivity by increasing PRLHR expression. **a**, **b** qPCR analysis of *PRLHR* mRNA expression levels in U87 (**a**) and U251 (**b**) cells treated with X5050 (100 µM for 48 h) (*n* = 3). **c**, **d** Western blot analysis of PRLHR protein expression levels in U87 (**c**) and U251 (**d**) cells treated with X5050 (100 µM for 48 h). **e**, **f** Immunofluorescence was used to detect γ-H2AX foci in U87 (**e**) and U251 (**f**) cells treated with X5050 (100 µM, 48 h) followed by irradiation (4 Gy) and incubation for 24 h (*n* = 3). Left panels: Representative images of γ-H2AX foci. Right panels: Quantification data. Scale bars, 50 μm. **g**, **h** Comet assay was performed to detect total DNA damage in U87 (**g**) and U251 (**h**) cells treated with X5050 (100 µM, 48 h) followed by irradiation (4 Gy, 24 h post-irradiation) or non-irradiation (*n* = 3). Left panels: Representative comet assay fluorescence images. Right panels: Quantification data (tail moment). Scale bars, 50 μm. **i**, **j** Clonogenic survival assay for U87 (**i**) and U251 (**j**) cells treated with X5050 (100 µM for 48 h) (*n* = 3). Left panels: Representative images of colonies. Middle panels: Survival fractions at different radiation doses, with curves fitted using the linear-quadratic (LQ) model; Right panels: Dose Enhancement Ratio (DER). **k**, **l** qPCR analysis of DNA repair-related gene expression changes in U87 (**k**) and U251 (**l**) cells treated with X5050 (100 µM for 48 h) (*n* = 3). *p < 0.05, **p < 0.01, ***p < 0.001, ****p < 0.0001 by Student’s t-test

X5050, a compound that selectively induces degradation of the REST protein, was employed as a potent REST inhibitor [36]. Zhang et al. demonstrated the antitumor effects of X5050 on human leukemia [37]. In our study, X5050 (100 µM for 48 h) effectively inhibits REST expression and increased *PRLHR* mRNA and protein levels in U87, U251, and LN229 cells (Fig. 6a–d and Fig. S6a–b). In addition, X5050 increased γ-H2AX foci formation and comet tail moments in these cells after irradiation, indicating elevated levels of DNA damage (Fig. 6e–h and Fig. S6c–d). To evaluate the radiosensitizing effect of PRLHR inhibition, we performed clonogenic assays. Notably, X5050 treatment alone significantly suppressed the plating efficiency (PE) of U87, U251, and LN229 cells (Fig. S6e–g), reflecting its potent single-agent cytotoxicity. After normalizing the survival fractions to the 0 Gy controls, we found that X5050 significantly enhanced the radiosensitivity of these GBM cell lines (Fig. 6i–j and Fig. S6h) and reduced the expression of several DNA repair-related genes (such as *FEN1, TYMS*, and *RAD51*) (Fig. 6k–l and Fig. S6i). These results indicate that REST downregulates PRLHR expression by binding to its promoter. Knockdown of *REST* or pharmacological inhibition using X5050 increases irradiation-induced DNA damage and enhances radiosensitivity in GBM cells.

### REST inhibitor enhances the therapeutic efficacy of radiotherapy in GBM in vivo

**Fig. 7:**
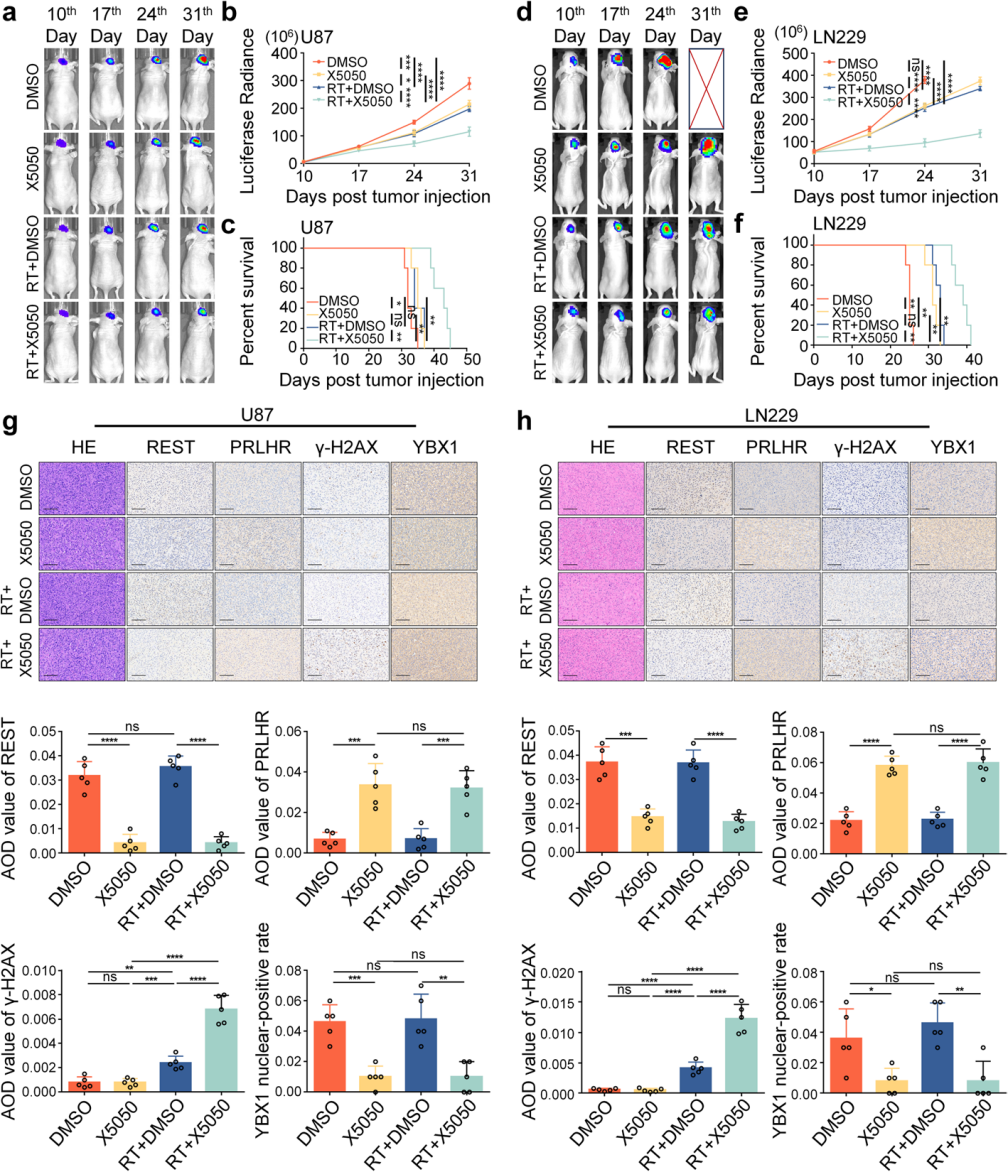
REST inhibitor enhances the therapeutic efficacy of radiotherapy in GBM in vivo. **a**-**f** Representative in vivo bioluminescence images (showing one mouse per group) monitoring tumor burden (**a**, **d**) (*n* = 5 per group), quantification of tumor bioluminescence radiance over time (**b**, **e**), and Kaplan–Meier survival analysis (**c**, **f**) of mice bearing orthotopic U87 (**a**-**c**) or LN229 (**d**-**f**) GBM intracranial tumors. **g**, **h** HE staining and IHC analysis of REST, PRLHR, γ-H2AX, and YBX1 protein levels in tumor tissues from the indicated groups in U87 (**g**) and LN229 (**h**) models. Upper panels: Representative HE and IHC staining images. Lower panels: Quantification of the indicated protein expression levels (*n* = 5). AOD, average optical density. Scale bar, 50 µm. *p < 0.05, **p < 0.01, ***p < 0.001, ****p < 0.0001

To further validate the translational potential of our findings and investigate their therapeutic relevance in GBM, we performed in vivo experiments using orthotopic GBM xenograft models established with U87 and LN229 cells. Ten days post-implantation, the mice were randomized into four treatment groups (*n* = 5): DMSO (control), X5050 monotherapy, Radiotherapy (RT) + DMSO, and RT + X5050. Tumor progression was monitored longitudinally via bioluminescence imaging. At the experimental endpoint, mice were euthanized, and tumor tissues were harvested for H&E staining and IHC analysis (Fig. S7a). Notably, the combination of RT and X5050 significantly suppressed GBM progression and markedly prolonged the overall survival of tumor-bearing mice compared to the control group, without significantly affecting mouse body weight (Fig. 7a–f and Fig. S7b–c).

To corroborate our cellular and molecular mechanisms in vivo, we performed IHC analysis on the excised tumor tissues. The results demonstrated that X5050 treatment effectively downregulated REST protein expression and upregulated PRLHR levels, irrespective of concurrent radiation. Furthermore, X5050 treatment resulted in a substantial reduction in the nuclear positivity of YBX1. Importantly, the combined RT and X5050 group exhibited the highest levels of γ-H2AX expression among all groups (Fig. 7g–h). Collectively, these in vivo data align with our in vitro observations, suggesting that X5050 upregulates PRLHR expression and inhibits the nuclear translocation of YBX1, thereby exacerbating radiation-induced DNA damage, and ultimately improves the therapeutic efficacy of radiotherapy in GBM.

## Discussion

**Fig. 8:**
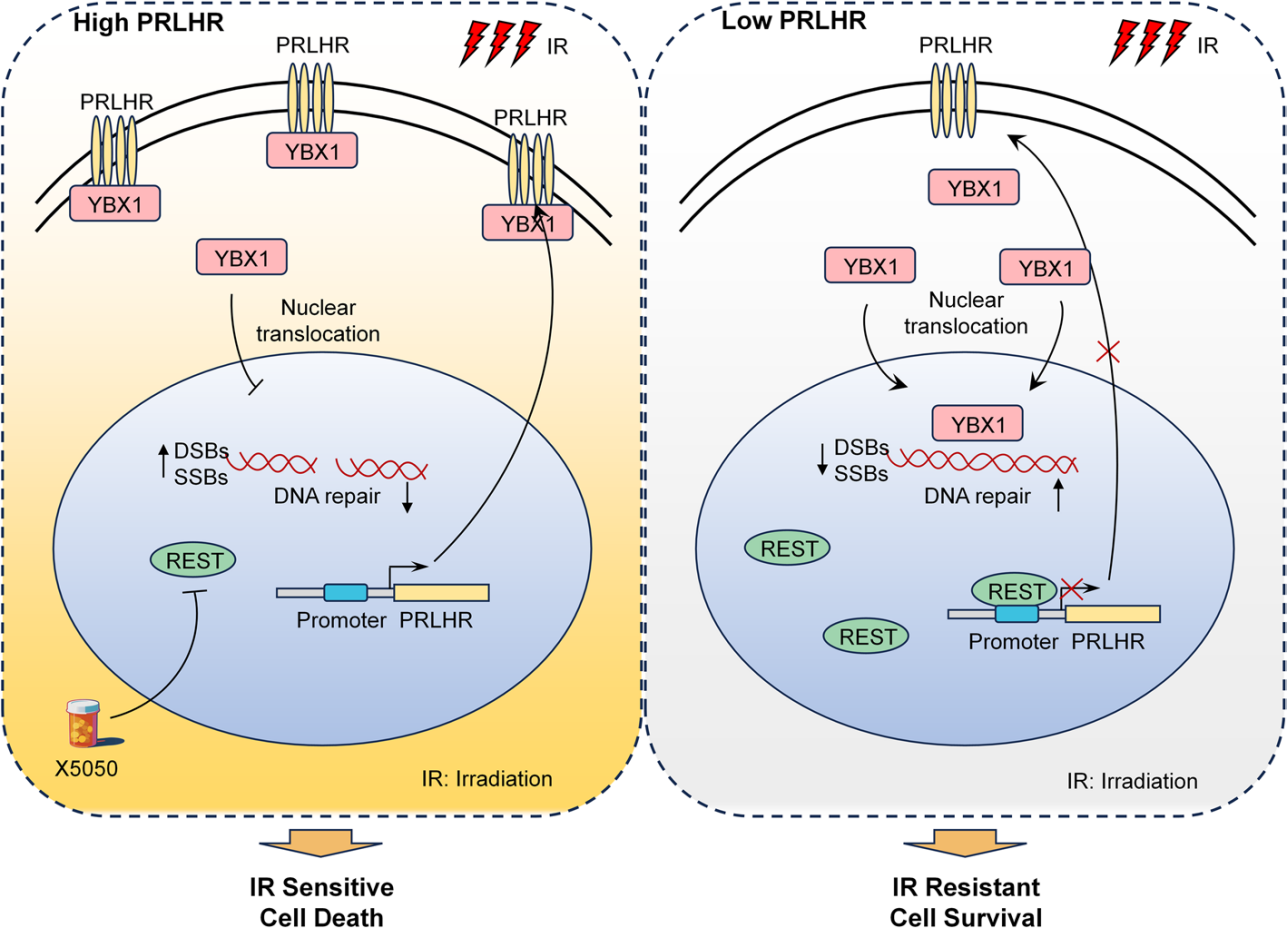
Schematic diagram illustrating the mechanism by which PRLHR enhances radiosensitivity in GBM cells

Radioresistance limits the therapeutic efficacy of radiotherapy in many cancers, including GBM. Although radiation-induced DNA damage can cause tumor cell death, DNA damage and repair are frequently modulated by intricate intrinsic cellular mechanisms, leading to radioresistance, tumor progression, and recurrence [9, 12, 38]. However, the genetic and molecular mechanisms regulating GBM radiosensitivity remain incompletely elucidated. In this study, we provide evidence that PRLHR plays a critical role in regulating GBM radiosensitivity. We demonstrated that patients with glioma who exhibit high PRLHR expression respond more favorably to radiotherapy. In GBM cells, *PRLHR* overexpression significantly enhanced radiosensitivity. Mechanistically, PRLHR directly interact with YBX1, inhibiting its nuclear translocation. This reduces nuclear YBX1 protein levels, thereby suppressing DNA repair; consequently, DNA damage accumulates, leading to increased radiosensitivity. We further showed that REST binds to the *PRLHR* promoter, reducing PRLHR protein expression, and ultimately conferring radioresistance in GBM cells. Treatment with the REST inhibitor X5050 reversed this effect by increasing PRLHR protein levels and enhancing GBM radiosensitivity (Fig. 8). Our findings demonstrate the significant role of PRLHR in regulating GBM radiosensitivity and highlight its potential as a novel therapeutic target to enhance response to radiotherapy and improve patient outcomes.

The normal function of PRLHR is primarily restricted to the hypothalamic region of the brain [16]. Although PRLHR expression and function in glioma and GBM have not been previously reported, elevated PRLHR levels have been identified as a protective prognostic factor in colorectal cancer [17]. Furthermore, studies have indicated that in lower-grade gliomas (LGG), PRLHR expression negatively correlates with immune cell infiltration and suppresses LGG progression [18, 19]. Interestingly, studies have shown that the prolactin receptor (PRLR), rather than the prolactin-releasing hormone receptor (PRLHR), has promoted the proliferation of GBM cells [39]. Within our glioma patient cohorts, PRLHR expression was significantly elevated in the long PFS group relative to the short PFS group; notably, higher PRLHR expression correlated with more favorable responses to radiotherapy (Fig. 1). Collectively, these findings support the notion that high PRLHR expression is associated with a favorable prognosis in gliomas, including GBM, and contributes to the regulation of tumor cell radiosensitivity. Importantly, our results further confirmed that PRLHR enhanced GBM radiosensitivity both in vitro and in vivo (Fig. 2).

DNA damage represent the most lethal form of damage inflicted by ionizing radiation on tumor cells, triggering a cascade of DNA damage responses that are crucial for tumor cell radiosensitivity [40, 41]. Consequently, over the past decades, enhancing tumor response to radiation through DNA damage pathways has been a major focus of radiotherapy research [42]. Our results demonstrate that *PRLHR* overexpression increased DNA damage and inhibited DNA repair pathways in GBM cells following radiation (Fig. 3 and Fig. S3), suggesting that PRLHR regulates GBM radiosensitivity by influencing DNA damage and DNA repair. Using IP-MS and co-IP experiments with U87 cell lysates, we identified a direct interaction between PRLHR and YBX1. YBX1 functions as a transcription factor for multiple drug resistance-associated genes and plays a substantial role in various DNA repair mechanisms [43, 44]. Previous studies have shown that YBX1 undergoes phosphorylation in response to DNA damage, facilitating its nuclear translocation and interaction with other proteins [45–47]. Studies have shown that nuclear translocation of YBX1 contributes to homologous recombination repair by promoting the transcription and expression of BRCA1 [35]. Furthermore, our results showed that *YBX1* overexpression reversed the *PRLHR* overexpression-induced increase in DNA damage, leading to reduced DNA damage in GBM cells post-radiation (Fig. 4i–n and Fig. S4e–g). This confirms the pivotal role of YBX1 in PRLHR-mediated regulation of GBM radiosensitivity: PRLHR binds to YBX1, inhibits its nuclear translocation, reduces nuclear YBX1 levels, suppresses DNA repair activity, and ultimately enhances the radiosensitivity of GBM cells.

REST, also known as neuron-restrictive silencer factor (NRSF), plays key roles in neuronal differentiation, axon growth, vesicle trafficking, and neurotransmitter release [48]. Studies have shown that REST expression is elevated in human GBM and medulloblastoma compared to adjacent normal tissues [49]. In our study, REST expression was significantly elevated in the short PFS GBM group relative to the long PFS group. REST binds to a 21‒23 bp repressor element (RE1) located in the promoters of over 2000 genes in the human genome. This binding epigenetically silences target genes [50, 51]. Previous studies have reported that PRLHR may be a downstream target of REST in uterine fibroids [20]. In this study, we confirmed that REST binds to the *PRLHR* promoter and negatively regulates its expression in GBM cells (Fig. 5b and c). Concurrently, *REST* knockdown increased PRLHR expression, subsequently inhibits the DNA repair pathway, and increased DNA damage. This ultimately enhanced the radiosensitivity of the GBM (Fig. 5d–o and Fig. S5a–e). These findings indicate that REST acts as an upstream regulator of PRLHR in GBM, modulating radiosensitivity by controlling PRLHR expression. X5050, an inhibitor of REST, induces selective degradation of the REST protein. It has demonstrated clinical efficacy in various brain disorders, including epilepsy [36, 52]. Beyond its significant independent anti-proliferative activity (as evidenced by the marked reduction in baseline plating efficiency), we verified that X5050 further enhances GBM radiosensitivity through the upregulation of PRLHR (Fig. 6 and Fig.S6), highlighting its multifaceted potential as both a potent monotherapy and a robust radiosensitizer. In vivo experiments further demonstrated that the combination treatment resulted in the greatest tumour response and showed that X5050 increased DNA damage in GBM following radiotherapy (Fig. 7a–f). These results suggest that X5050 may serve as a therapeutic agent for improving the outcome of GBM radiotherapy.

This study has several limitations that warrant resolution in subsequent research. The limited number of glioma patients who received radiotherapy enrolled in the present study precluded us from performing stratified analysis according to the WHO grading system for gliomas. Therefore, in future investigations, we will expand the sample size and conduct stratified analyses stratified by WHO grade to enhance the robustness of our findings. Additionally, to overcome the inherent limitations of established cell lines, we plan to employ patient-derived xenograft (PDX) models in future studies to further validate our results in a more clinically relevant setting. Furthermore, the specific molecular mechanism whereby PRLHR inhibits the nuclear localization of YBX1 remains not fully elucidated in this study. Subsequent research will focus on clarifying the precise mechanism through which PRLHR interacts with YBX1 to abrogate its nuclear translocation, as well as identifying relevant molecular targets for potential therapeutic intervention.

In summary, based on the hypothesis that identifying novel molecular axes could overcome radioresistance in GBM, we investigated the functional role and upstream regulation of PRLHR in this study. By integrating analyses of clinical patient cohorts with comprehensive in vitro mechanistic assays, transcriptomic analyses, and in vivo preclinical mouse models, our study establishes that PRLHR enhances the radiosensitivity of GBM by suppressing YBX1 nuclear translocation and impairing DNA damage repair, thereby promoting the accumulation of DNA lesions and augmenting the radiosensitive response. Moreover, we further show that REST negatively modulates PRLHR protein expression by binding to the PRLHR promoter, thereby modulating GBM radiosensitivity. Notably, pharmacological intervention using the REST inhibitor X5050 significantly enhanced the therapeutic efficacy of radiotherapy in remarkably suppressing tumor growth in vivo. Collectively, our findings position PRLHR as a promising radiosensitization target in GBM and underscore its potential to inform the development of more effective and precision-guided cancer therapies.

## Materials and methods

### Patient sample collection

A total of 144 patients were enrolled from those who underwent surgical resection followed by postoperative radiotherapy in the Department of Neurosurgery, The First Affiliated Hospital of Zhengzhou University, Zhengzhou, China. The inclusion criteria were as follows: (1) glioma diagnosed by neuroimaging and pathology; (2) first-onset glioma with initial surgical resection; (3) Recurrence after postoperative radiotherapy; (4) no other nervous system disease, and (5) age > 18 years. The exclusion criteria were as follows: (1) cerebral hernia or tumor-associated stroke before surgery, (2) severe systemic diseases, (3) mental disorders, and (4) age < 18 years. All enrolled patients were followed up via phone or hospital visits. The clinical data of 144 patients with glioma are shown in Table S1. This study was approved by the College Accreditation Committee of Zhengzhou University and conducted in accordance with the Declaration of Helsinki. All samples used in this study were obtained with the informed consent of patients. Human glioma specimens were used in accordance with the principles set by the Ethics Committee of Zhengzhou University (Approval No. 2019-KY-0032).

### RNA sequencing (RNA-seq) analysis

We utilized TRIzol reagent (Beyotime, R0017S) to isolate total RNA from glioma tissues (central tumor core) and U87/U251 cells. To ensure high quality, only core regions confirmed by histopathology and navigation to be non-necrotic were selected. RNA integrity was validated using a Bioanalyzer. Starting with 1 µg of total RNA, we captured polyadenylated transcripts using oligo(dT) beads or depleted rRNA with RiboZero. The fragmented RNA underwent reverse transcription using SuperScript IV and random primers to yield double-stranded cDNA. Library preparation, including end-polishing and adapter attachment, was performed with the NEBNext Ultra II kit. The resulting libraries were quantified, equimolarly mixed, and sequenced on an Illumina NovaSeq 6000 (50 bp paired-end; 30 M reads/sample). Sequence alignment to the GRCh38 genome was executed via STAR/Hisat2 after quality assessment. Differential expression was analyzed using DESeq2, where genes exhibiting an adjusted *P*-value < 0.05 were categorized as significant DEGs. To visualize the DEGs, a volcano plot was generated using the ggplot2 package in R. Furthermore, Gene Ontology (GO) enrichment analysis of the identified DEGs was conducted using the clusterProfiler R package to identify significantly enriched biological processes, cellular components, and molecular functions.

### Cell culture and reagents

Human GBM cells (U87, U251 and LN229; Hanghai QuiCell Biotechnology Co., Ltd.) were grown in DMEM (Sigma, Cat. No. D5796) containing 10% FBS. Maintenance occurred in a 37 °C, 5% CO₂ humidified incubator. We confirmed cell identity through STR profiling and monitored for mycoplasma presence on a quarterly basis. Regarding reagents, X5050 (HY-136833, MedChemExpress) was reconstituted in DMSO and preserved at −80 °C until use.

### Lentiviral plasmids, lentivirus transduction, and siRNA-mediated knockdown

FUB-MCS-P2A-EGFP-T2A-puro, pMD2.G, and psPAX2 plasmids were provided by Dr. Chenglin Zhang (Sino-British Research Center for Molecular Oncology, Zhengzhou University, China). For gene overexpression, PRLHR and Y-box-binding protein-1 (YBX1) nucleotides were ligated into the lentiviral plasmid vector FUB-MCS-P2A-EGFP-T2A-puro. A control lentivirus expressing luciferase was obtained from TSINGKE Biotechnology Co., Ltd. (Beijing, China). All constructs were verified using Sanger sequencing.

To establish GBM cell lines stably overexpressing the target protein, U87, U251 and LN229 cells were infected with the recombinant lentivirus, constructed as described in the previous section, for 72 h. Lentivirus-infected GBM cells were selected by continuous culture in 5 μg/mL puromycin. Infection efficiency was determined by reverse transcription quantitative real-time polymerase chain reaction (RT-qPCR) and western blotting.

To knock down the repressor element-1 binding transcription factor (REST) expression, a sequence-specific siRNA targeting human *REST* was used. The siRNA (si*REST*-1, 5'-GAGCGAGTCTACAAGTGTA-3'; si*REST*-2, 5'-GCAGTAAGCCTCCTCAGAA-3') was synthesized by Ribobio Co., Ltd. (Guangzhou, China). U87 and U251 cells were transfected using the JetPRIME transfection reagent (Polyplus).

### Irradiation

Irradiation was performed using the X-RAD 225 (Precision X-Ray, North Branford, CT, USA) system at a dose of 2 Gy/min. For GBM cells, culture dishes were placed approximately 50 cm directly beneath the irradiation probe, and irradiation duration was determined based on the prescribed radiation dose. For animal models, BALB/c nude mice were anesthetized using continuous inhalation of isoflurane (2% induction, 1.5% maintenance) and positioned such that the apex of each tumor was centered within the aperture of the secondary collimator, while the remaining body was shielded to limit radiation exposure.

### RT-qPCR assay

Extraction of total RNA was performed through the TRIzol method, with quantification and quality assessment conducted via spectrophotometric analysis. For cDNA preparation, 1 μg of the isolated RNA was reverse-transcribed using a PrimeScript RT kit. Subsequent qPCR was executed on a QuantStudio platform, utilizing a reaction mixture of SYBR Green Master Mix, specific primers (0.2 μM), and 2 μL of cDNA. The thermal cycling profile included an initial denaturation at 95 °C for 30 s, followed by 40 cycles (95 °C for 5 s and 60 °C for 30 s) and a final melt curve step. Target gene levels were normalized to *GAPDH* and determined using the 2^−ΔΔCt^ comparative method. All primer information is detailed in Table S2.

### Western blotting assay

To extract total protein, cells were harvested in RIPA lysis buffer supplemented with a protease inhibitor cocktail. Protein levels were quantified using a BCA assay kit (Beyotime, China). Equivalent amounts of protein samples were resolved by SDS-PAGE and subsequently electrotransferred onto PVDF membranes. After a 1-h blocking step with 5% skim milk in TBST at room temperature, the membranes were probed overnight at 4 °C with the following primary antibodies: PRLHR (1:500, ABclonal, A10479), GAPDH (1:50000, Proteintech, 60004-1), YBX1 (1:5000, H00004904-M02; ThermoFisher), Lamin A/C (1:5000, Proteintech, 10298-2), p-H2A.X (Ser139; 1:1000, CST, 2577S), and Tubulin (1:3000, Proteintech, 80762-1). Following three washes, the membranes were incubated for 1 h at room temperature with HRP-labeled secondary antibodies (1:5000, Proteintech, RGAR001 or RGAM001). Immunoreactive bands were developed using enhanced chemiluminescence (ECL) reagents and captured with a digital imaging system.

### Cell viability assay

Cell proliferation was monitored using the Cell Counting Kit-8 (CCK-8, MedChemExpress, HY-K0301). Briefly, logarithmic-phase cells were plated in 96-well microplates at a density of 1 × 10^3^–5 × 10^3^ per well (100 μL per well) and maintained at 37 °C under 5% CO₂ for 24 h. At the scheduled time points (24, 48, and 72 h), each well was supplemented with 10 μL of CCK-8 reagent. Following an additional 2-h incubation period, the optical density (OD) was measured at 450 nm using a Bio-Rad microplate reader. All assays were conducted in triplicate using independent biological samples to ensure reproducibility.

### Colony formation assay

Cells were seeded at low density in six-well plates. After 24 h, cultures were treated with irradiation, X5050, or both, and incubated for 7‒14 days. Colonies (with > 50 cells) were fixed using methanol: acetic acid (3:1), stained with 0.5% crystal violet, and counted macroscopically. The survival fraction (SF) and dose enhancement ratio (DER) were calculated as follows:

Cell survival data were analyzed using the linear-quadratic (L-Q) model. The survival fraction (SF) for each irradiated sample was calculated using the following equation: SF = $\frac{\mathrm{Colonies}\;\mathrm{Counted}/\mathrm{Cells}\;\mathrm{Seeded}\;(\mathrm{irradiated})}{\mathrm{PE}}$, where PE (plating efficiency) represents the colony yield per cell in the non-irradiated control group. To determine the radiobiological parameters, the natural logarithm of the survival fraction (ln(SF)) was fitted against the radiation dose (D) using a second-order polynomial regression: ln(SF) = − (αD + βD^2^). The parameters α(Gy^−1^) and β(Gy^−2^) were calculated using GraphPad Prism 9.0. To quantify the radiosensitizing effect, the dose enhancement ratio (DER) was calculated based on the dose required to achieve a survival fraction of 10% (D_10_). The D_10_ for each group was calculated from the L-Q parameters as follows: D_10_ = $\frac{-\upalpha +\sqrt{\upalpha^{\wedge} 2-4\upbeta \mathrm{l}\mathrm{n}(0.1)}}{2\upbeta }$. The DER was then determined using the formula: $D\mathrm{ER}=\frac{\mathrm D10(\mathrm{Radiation}\;\mathrm{alone})}{\mathrm D10(\mathrm{Treatment}+\mathrm{Radiation})}$. Importantly, to ensure rigorous statistical evaluation, the curve fitting and subsequent DER calculation were performed independently for each of the three biological replicates (*n* = 3). The final DER results are reported as the mean ± standard deviation (SD) derived from these independent measurements.

A DER value > 1 indicates increased radiosensitivity; a DER value = 1 denotes no radiosensitizing effect; and a DER value < 1 represents decreased radiosensitivity, i.e., radioresistance.

### Hematoxylin and eosin (H&E) staining and immunohistochemistry assay

Clinical specimens from 61 glioma patients (distributed as WHO Grade 2: *n* = 21, Grade 3: *n* = 10, and Grade 4: *n* = 30) along with brain tissues from 20 mouse models were processed for H&E and IHC staining. For the H&E procedure, after dewaxing and rehydration, slides were stained with hematoxylin for 5 min and eosin for 2 min. Post-staining, sections were dehydrated, cleared via xylene, and sealed using neutral resin. Regarding IHC, heat-induced antigen retrieval was carried out in a 6.0 pH citrate buffer. Following the inhibition of endogenous peroxidase and non-specific sites, the slides were exposed to primary antibodies (PRLHR, 1:100, ABclonal, A10479; REST, 1:500, Proteintech, 22242-1; p-H2A.X, 1:500, CST, 2577S; and YBX1, 1:500, H00004904-M02; ThermoFisher) at 4 °C for an overnight period. Signal detection was achieved using HRP-labeled secondary antibodies and DAB staining, with hematoxylin as a counterstain. Microscopic imaging was used to capture the results. Quantification of IHC staining was performed using ImageJ software (NIH, Bethesda, MD, USA). The average optical density (AOD) was calculated from at least five randomly selected fields of view per sample using the formula: AOD = integrated optical density (IOD)/area of positive staining.

### Intracranial mouse model

Female BALB/c nude mice (4–6 weeks old, weight 16–22 g) were purchased from Vital River Laboratory Animal Technology. Mice were housed in a specific pathogen-free (SPF) environment under a 12-h light/dark cycle with ad libitum access to food and water. Following the protocol described by Piells et al. [53], an orthotopic intracranial tumor model in mice was established. Briefly, luciferase-expressing U87 or LN229 cells were adjusted to a concentration of 2 × 10^7^ cells/ml. After anesthetizing BALB/c nude mice with inhaled isoflurane (2% induction, 1.5% maintenance), the animals were secured in a stereotactic head frame. Following exposure of the skull, a 1-mm-diameter hole was drilled in the skull, and 5 μL of the cell suspension was slowly injected into the brain using a microsyringe. After suturing the scalp, mice were transferred to a heating pad for recovery. On post-intracranial injection day 10 (PID 10), tumor establishment was confirmed via a bioluminescence system (IVIS Spectrum; PerkinElmer). Mice with successfully established tumors were randomly assigned to designated experimental subgroups, and the irradiation group received radiation therapy (RT) initiated on the same day (PID 10), administered as 5 fractions of 2 Gy (total cumulative dose: 10 Gy). Tumor volume was serially quantified once weekly for the duration of the study. X5050 was dissolved in DMSO and administered to the mice via intraperitoneal injection at a dose of 0.25 mg/kg (twice weekly). All procedures involving animal models were performed in compliance with ethical standards and were approved by the institutional ethics committee (Approval No. ZZU-LAC20231201).

### Immunofluorescent assay

After 15 min of fixation (4% PFA) and 10 min of permeabilized treatment (0.1% Triton X-100), coverslip-grown cells were blocked in 5% BSA. We incubated the samples overnight at 4 °C with the following primary antibodies: phospho-histone H2A.X (1:500, CST, 2577S) and YBX1 (1:200, H00004904-M02; ThermoFisher). Detection was performed using a light-sensitive incubation (1 h) with Alexa Fluor 555-labeled secondary IgG (1:500, Beyotime). The nuclei were then counterstained using DAPI before sealing with antifade media. Representative images were captured on a Leica confocal station using a 100 ×/1.3 NA oil objective under constant acquisition settings.

### Comet assay

To evaluate total DNA damage, including single-strand breaks (SSBs), double-strand breaks (DSBs), and alkali-labile sites, we conducted comet assays using a commercial kit (KeyGEN Biotech). Briefly, target cells were embedded in 1% low-melting-point (LMP) agarose and then immobilized on pre-treated slides. Following refrigeration at 4 °C for 10 min to allow for gelation, the slides were submerged in chilled lysis solution for an hour. For the purpose of DNA unwinding, the slides were soaked in an alkaline buffer for 20 min. Electrophoretic separation was executed at 25 V and 300 mA for 20 min in the same alkaline environment. After a neutralization step (0.4 M Tris, pH 7.5), nuclei were stained with propidium iodide (PI) and captured via fluorescence microscopy. The extent of DNA damage was evaluated using CASP (Comet Assay Software Project) software.

### Immunoprecipitation and mass spectrometry

PHLHR-overexpressing U87 cells were seeded in 10-cm dishes, lysed, and immunoprecipitated using anti-IgG or anti-PRLHR antibodies. Successful immunoprecipitation of PRLHR was verified by western blotting. The immunoprecipitated proteins were analyzed using liquid chromatography-tandem mass spectroscopy performed by Jingjie PTM Biolabs (Hangzhou, China).

### Co-immunoprecipitation (co-IP) assay

To investigate protein–protein interactions, U87, U251 and LN229 cells overexpressing *PRLHR* were cultured in 10-cm vessels and subsequently lysed with BOSTER lysis buffer (16H17B08) containing a CWBIO protease inhibitor cocktail (CW2200S). The resulting lysates were subjected to immunoprecipitation at 4 °C overnight using either rabbit anti-PRLHR pAb (A10478; Abclone) or mouse anti-YBX1 mAb (H00004904-M02; ThermoFisher). For negative controls, rabbit (AC005) and mouse (AC011) IgG isotypes were employed. After capturing the complexes with protein A/G beads (rotating for 3 h at 4 °C), the beads were washed and centrifuged five times with chilled, inhibitor-supplemented PBS. Finally, the bound proteins were eluted in 10% SDS buffer and characterized via western blot analysis.

### Nuclear and cytoplasmic protein extraction

To isolate nuclear and cytoplasmic components, we utilized a specialized extraction kit from Beyotime (P0027, Shanghai, China) according to the provided protocol. In brief, treated cells were rinsed with PBS and subjected to lysis in a hypotonic environment on ice for 15 min. Following centrifugation at 500 × g (5 min, 4 °C), the resulting supernatant was harvested as the cytoplasmic fraction. The remaining nuclear pellets were then washed, reconstituted in high-salt buffer, and agitated via vortexing for 30 min at 4 °C. Finally, clear nuclear lysates were obtained by centrifugation at 12,000 × g for 10 min.

### Chromatin immunoprecipitation assay

Cell samples underwent a 10-min fixation in 1% formaldehyde before being quenched by glycine and homogenized in lysis buffer. Chromatin shearing was performed using a sonicator to yield 200–500 bp fragments. These sheared samples were then incubated with Protein A/G beads pre-bound to anti-REST or control IgG antibodies at 4 °C overnight. The beads were subsequently washed with salt buffers of varying stringency before the elution of the complexes. Thermal decrosslinking occurred at 65 °C for 4 h, followed by a Proteinase K digestion step. The resulting DNA was purified for subsequent PCR-based analysis.

### Statistical analysis

Data from three independent trials are reported as mean ± SD. Normality was assessed using Shapiro–Wilk tests. Normally distributed data were analyzed by two-tailed unpaired t-tests, whereas nonparametric tests were used otherwise. One-way or two-way ANOVA was utilized for comparisons involving more than two groups. Spearman’s rank correlation identified the association between *REST* and *PRLHR* mRNA levels. Survival rates were estimated via Kaplan–Meier curves and the log-rank test. To evaluate survival-related indicators, Cox proportional hazards regression models were established across the FAHZZU, CGGA325, CGGA693, and GSE16011 datasets, adjusting for age, sex, PRLHR expression, resection extent, 1p/19q status, and chemotherapy. Results are presented as HRs with 95% CIs. Statistical significance was designated at *p* < 0.05.

## Supplementary Information

Supplementary Material 1.

## Data Availability

All data needed to interpret the results are presented in this article and its supplementary information files. The raw RNA-seq data generated in this study have been deposited into the Genome Sequence Archive (GSA) at the National Genomics Data Center (CNCB-NGDC), Beijing Institute of Genomics (China National Center for Bioinformation), Chinese Academy of Sciences, under the accession number HRA006184 and HRA017387. Data are available to qualified researchers upon reasonable request to Dr. Zhenyu Zhang (fcczhangzy1@zzu.edu.cn). Access requires a formal proposal and approval from institutional ethics and data management committees.

## Abbreviations

GBM: Glioblastoma Multiforme
LGG: Lower-Grade Gliomas
PRLHR: Prolactin-releasing Hormone Receptor
YBX1: Y-box-binding Protein-1
REST: Repressor Element-1 Binding Transcription Factor
DSBs: Double-Strand Breaks
DER: Dose Enhancement Ratio
SSBs: Single-strand breaks
IDH: Isocitrate Dehydrogenase
TME: Tumor Microenvironment
ROS: Reactive Oxygen Species
PrRP: Prolactin-releasing Peptide
DEGs: Differentially Expressed Genes
STR: Short Tandem Repeat
DMSO: Dimethyl Sulfoxide
RT-qPCR: Reverse Transcription Quantitative Real-time Polymerase Chain Reaction
GO: Gene Ontology
IHC: Immunohistochemical
H&E: Hematoxylin and Eosin
DAPI: 4’,6-Diamidino-2-phenylindole Dihydrochloride
Co-IP: Co-immunoprecipitation
SD: Standard Deviation
PFS: Progression-Free Survival
OS: Overall Survival
*ZWINT*: ZW10 Interacting Protein 1
*FEN1*: Flap Endonuclease 1
*TYMS*: Thymidylate Synthetase
*POLA1*: Polymerase DNA Directed Alpha 1
*PCNA*: Proliferating Cell Nuclear Antigen
MS: Mass Spectrometry

## References

[1] Price M, Ballard C, Benedetti J, Neff C, Cioffi G, Waite KA, et al. CBTRUS statistical report: primary brain and other central nervous system tumors diagnosed in the United States in 2017-2021. Neuro Oncol. 2024;26(Supplement_6):vi1–85. 10.1093/neuonc/noae145. 39371035 10.1093/neuonc/noae145 PMC11456825

[2] Louis DN, Perry A, Wesseling P, Brat DJ, Cree IA, Figarella-Branger D, et al. The 2021 WHO classification of tumors of the central nervous system: a summary. Neuro Oncol. 2021;23(8):1231–51. 10.1093/neuonc/noab106. 34185076 10.1093/neuonc/noab106 PMC8328013

[3] Wen PY, Weller M, Lee EQ, Touat M, Khasraw M, Rahman R, et al. Glioblastoma in adults: a Society for Neuro-Oncology (SNO) and European Society of Neuro-Oncology (EANO) consensus review on current management and future directions. Neuro Oncol. 2025;27(11):2751–88. 10.1093/neuonc/noaf177. 40827022 10.1093/neuonc/noaf177 PMC12908567

[4] Schaff LR, Mellinghoff IK. Glioblastoma and other primary brain malignancies in adults: a review. JAMA. 2023;329(7):574–87. 10.1001/jama.2023.0023. 36809318 10.1001/jama.2023.0023 PMC11445779

[5] Nakano T, Akamatsu K, Tsuda M, Tujimoto A, Hirayama R, Hiromoto T, et al. Formation of clustered DNA damage in vivo upon irradiation with ionizing radiation: visualization and analysis with atomic force microscopy. Proc Natl Acad Sci U S A. 2022;119(13):e2119132119. 10.1073/pnas.2119132119. 35324325 10.1073/pnas.2119132119 PMC9060515

[6] Barker HE, Paget JT, Khan AA, Harrington KJ. The tumour microenvironment after radiotherapy: mechanisms of resistance and recurrence. Nat Rev Cancer. 2015;15(7):409–25. 10.1038/nrc3958. 26105538 10.1038/nrc3958 PMC4896389

[7] Erasimus H, Gobin M, Niclou S, Van Dyck E. DNA repair mechanisms and their clinical impact in glioblastoma. Mutat Res Rev Mutat Res. 2016;769:19–35. 10.1016/j.mrrev.2016.05.005. 27543314 10.1016/j.mrrev.2016.05.005

[8] Carlos-Reyes A, Muniz-Lino MA, Romero-Garcia S, Lopez-Camarillo C, Hernandez-de la Cruz ON. Biological adaptations of tumor cells to radiation therapy. Front Oncol. 2021;11:718636. 10.3389/fonc.2021.718636. 34900673 10.3389/fonc.2021.718636 PMC8652287

[9] Huang R, Zhou PK. DNA damage repair: historical perspectives, mechanistic pathways and clinical translation for targeted cancer therapy. Signal Transduct Target Ther. 2021;6(1):254. 10.1038/s41392-021-00648-7. 34238917 10.1038/s41392-021-00648-7 PMC8266832

[10] Nickoloff JA, Sharma N, Taylor L. Clustered DNA double-strand breaks: biological effects and relevance to cancer radiotherapy. Genes (Basel). 2020. 10.3390/genes11010099. 31952359 10.3390/genes11010099 PMC7017136

[11] Bernier J, Hall EJ, Giaccia A. Radiation oncology: a century of achievements. Nat Rev Cancer. 2004;4(9):737–47. 10.1038/nrc1451. 15343280 10.1038/nrc1451

[12] Wu Y, Song Y, Wang R, Wang T. Molecular mechanisms of tumor resistance to radiotherapy. Mol Cancer. 2023;22(1):96. 10.1186/s12943-023-01801-2. 37322433 10.1186/s12943-023-01801-2 PMC10268375

[13] Burko P, D’Amico G, Miltykh I, Scalia F, Conway de Macario E, Macario AJL, et al. Molecular pathways implicated in radioresistance of glioblastoma multiforme: what is the role of extracellular vesicles? Int J Mol Sci. 2023. 10.3390/ijms24054883. 36902314 10.3390/ijms24054883 PMC10003080

[14] Matsui JK, Perlow HK, Ritter AR, Upadhyay R, Raval RR, Thomas EM, et al. Small molecules and immunotherapy agents for enhancing radiotherapy in glioblastoma. Biomedicines. 2022. 10.3390/biomedicines10071763. 35885067 10.3390/biomedicines10071763 PMC9313399

[15] Mittal A, Nenwani M, Sarangi I, Achreja A, Lawrence TS, Nagrath D. Radiotherapy-induced metabolic hallmarks in the tumor microenvironment. Trends Cancer. 2022;8(10):855–69. 10.1016/j.trecan.2022.05.005. 35750630 10.1016/j.trecan.2022.05.005

[16] Ohshima K, Morii E. Metabolic reprogramming of cancer cells during tumor progression and metastasis. Metabolites. 2021. 10.3390/metabo11010028. 33401771 10.3390/metabo11010028 PMC7824065

[17] Chen Y, Li Z, Dong Z, Beebe J, Yang K, Fu L, et al. 14-3-3σ contributes to radioresistance by regulating DNA repair and cell cycle via PARP1 and CHK2. Mol Cancer Res. 2017;15(4):418–28. 10.1158/1541-7786.MCR-16-0366. 28087741 10.1158/1541-7786.MCR-16-0366 PMC5380477

[18] Goffart N, Lombard A, Lallemand F, Kroonen J, Nassen J, Di Valentin E, et al. CXCL12 mediates glioblastoma resistance to radiotherapy in the subventricular zone. Neuro Oncol. 2017;19(1):66–77. 10.1093/neuonc/now136. 27370398 10.1093/neuonc/now136 PMC5193023

[19] Rahman M, Hasan MR. Cancer metabolism and drug resistance. Metabolites. 2015;5(4):571–600. 10.3390/metabo5040571. 26437434 10.3390/metabo5040571 PMC4693186

[20] Varghese BV, Koohestani F, McWilliams M, Colvin A, Gunewardena S, Kinsey WH, et al. Loss of the repressor REST in uterine fibroids promotes aberrant G protein-coupled receptor 10 expression and activates mammalian target of rapamycin pathway. Proc Natl Acad Sci U S A. 2013;110(6):2187–92. 10.1073/pnas.1215759110. 23284171 10.1073/pnas.1215759110 PMC3568308

[21] Talbot F, Feetham CH, Mokrosinski J, Lawler K, Keogh JM, Henning E, et al. A rare human variant that disrupts GPR10 signalling causes weight gain in mice. Nat Commun. 2023;14(1):1450. 10.1038/s41467-023-36966-3. 36922513 10.1038/s41467-023-36966-3 PMC10017677

[22] Yin W, Jiang X, Tan J, Xin Z, Zhou Q, Zhan C, et al. Development and validation of a tumor mutation burden-related immune prognostic model for lower-grade glioma. Front Oncol. 2020;10:1409. 10.3389/fonc.2020.01409. 32974146 10.3389/fonc.2020.01409 PMC7468526

[23] Liu Y, Xiang J, Peng G, Shen C. PRLHR immune genes associated with tumor mutation burden can be used as prognostic markers in patients with gliomas. Front Oncol. 2022;12:620190. 10.3389/fonc.2022.620190. 35800054 10.3389/fonc.2022.620190 PMC9253814

[24] Geczi D, Klekner A, Balogh I, Penyige A, Szilagyi M, Virga J, et al. Identification of deregulated miRNAs and mRNAs involved in tumorigenesis and detection of glioblastoma patients applying next-generation RNA sequencing. Pharmaceuticals (Basel). 2025. 10.3390/ph18030431. 40143207 10.3390/ph18030431 PMC11944724

[25] Wu Q, Yin X, Zhao W, Xu W, Chen L. Downregulation of SFRP2 facilitates cancer stemness and radioresistance of glioma cells via activating Wnt/beta-catenin signaling. PLoS One. 2021;16(12):e0260864. 10.1371/journal.pone.0260864. 34852024 10.1371/journal.pone.0260864 PMC8635357

[26] Tang T, Wang LX, Yang ML, Zhang RM. lncRNA TPTEP1 inhibits stemness and radioresistance of glioma through miR-106a-5p-mediated P38 MAPK signaling. Mol Med Rep. 2020;22(6):4857–67. 10.3892/mmr.2020.11542. 33173989 10.3892/mmr.2020.11542 PMC7646932

[27] Wu X, Li C, Wang Z, Zhang Y, Liu S, Chen S, et al. A bioinformatic analysis study of m(7)G regulator-mediated methylation modification patterns and tumor microenvironment infiltration in glioblastoma. BMC Cancer. 2022;22(1):729. 10.1186/s12885-022-09791-y. 35788194 10.1186/s12885-022-09791-y PMC9251941

[28] Lu CH, Wei ST, Liu JJ, Chang YJ, Lin YF, Yu CS, et al. Recognition of a novel gene signature for human glioblastoma. Int J Mol Sci. 2022. 10.3390/ijms23084157. 35456975 10.3390/ijms23084157 PMC9029857

[29] Orlando UD, Castillo AF, Medrano MAR, Solano AR, Maloberti PM, Podesta EJ. Acyl-CoA synthetase-4 is implicated in drug resistance in breast cancer cell lines involving the regulation of energy-dependent transporter expression. Biochem Pharmacol. 2019;159:52–63. 10.1016/j.bcp.2018.11.005. 30414939 10.1016/j.bcp.2018.11.005

[30] Brizuela L, Ader I, Mazerolles C, Bocquet M, Malavaud B, Cuvillier O. First evidence of sphingosine 1-phosphate lyase protein expression and activity downregulation in human neoplasm: implication for resistance to therapeutics in prostate cancer. Mol Cancer Ther. 2012;11(9):1841–51. 10.1158/1535-7163.MCT-12-0227. 22784711 10.1158/1535-7163.MCT-12-0227

[31] Cheraghi-Shavi T, Jalal R, Minuchehr Z. TGM2, HMGA2, FXYD3, and LGALS4 genes as biomarkers in acquired oxaliplatin resistance of human colorectal cancer: a systems biology approach. PLoS One. 2023;18(8):e0289535. 10.1371/journal.pone.0289535. 37535601 10.1371/journal.pone.0289535 PMC10399784

[32] Zhou K, Liu Y, Zhao Z, Wang Y, Huang L, Chai R, et al. ABCC8 mRNA expression is an independent prognostic factor for glioma and can predict chemosensitivity. Sci Rep. 2020;10(1):12682. 10.1038/s41598-020-69676-7. 32728190 10.1038/s41598-020-69676-7 PMC7391768

[33] Ramos AR, Ghosh S, Dedobbeleer M, Robe PA, Rogister B, Erneux C. Lipid phosphatases SKIP and SHIP2 regulate fibronectin-dependent cell migration in glioblastoma. FEBS J. 2019;286(6):1120–35. 10.1111/febs.14769. 30695232 10.1111/febs.14769

[34] Sancar A, Lindsey-Boltz LA, Unsal-Kacmaz K, Linn S. Molecular mechanisms of mammalian DNA repair and the DNA damage checkpoints. Annu Rev Biochem. 2004;73:39–85. 10.1146/annurev.biochem.73.011303.073723. 15189136 10.1146/annurev.biochem.73.011303.073723

[35] Sun X, Gao C, Xu X, Li M, Zhao X, Wang Y, et al. FBL promotes cancer cell resistance to DNA damage and BRCA1 transcription via YBX1. EMBO Rep. 2023;24(9):e56230. 10.15252/embr.202256230. 37489617 10.15252/embr.202256230 PMC10481664

[36] Charbord J, Poydenot P, Bonnefond C, Feyeux M, Casagrande F, Brinon B, et al. High throughput screening for inhibitors of REST in neural derivatives of human embryonic stem cells reveals a chemical compound that promotes expression of neuronal genes. Stem Cells. 2013;31(9):1816–28. 10.1002/stem.1430. 23712629 10.1002/stem.1430

[37] Zhang Y, Chen K, Tang SC, Cai Y, Nambu A, See YX, et al. Super-silencer perturbation by EZH2 and REST inhibition leads to large loss of chromatin interactions and reduction in cancer growth. Nat Struct Mol Biol. 2025;32(1):137–49. 10.1038/s41594-024-01391-7. 39304765 10.1038/s41594-024-01391-7 PMC11746141

[38] Sulman EP, Ismaila N, Armstrong TS, Tsien C, Batchelor TT, Cloughesy T, et al. Radiation therapy for glioblastoma: American Society of Clinical Oncology clinical practice guideline endorsement of the American Society for Radiation Oncology guideline. J Clin Oncol. 2017;35(3):361–9. 10.1200/JCO.2016.70.7562. 27893327 10.1200/JCO.2016.70.7562

[39] Asad AS, Candia AJN, Gonzalez N, Zuccato CF, Abt A, Orrillo SJ, et al. Prolactin and its receptor as therapeutic targets in glioblastoma multiforme. Sci Rep. 2019;9(1):19578. 10.1038/s41598-019-55860-x. 31862900 10.1038/s41598-019-55860-x PMC6925187

[40] Huang RX, Zhou PK. DNA damage response signaling pathways and targets for radiotherapy sensitization in cancer. Signal Transduct Target Ther. 2020;5(1):60. 10.1038/s41392-020-0150-x. 32355263 10.1038/s41392-020-0150-x PMC7192953

[41] Santivasi WL, Xia F. Ionizing radiation-induced DNA damage, response, and repair. Antioxid Redox Signal. 2014;21(2):251–9. 10.1089/ars.2013.5668. 24180216 10.1089/ars.2013.5668

[42] Deycmar S, Faccin E, Kazimova T, Knobel PA, Telarovic I, Tschanz F, et al. The relative biological effectiveness of proton irradiation in dependence of DNA damage repair. Br J Radiol. 2020;93(1107):20190494. 10.1259/bjr.20190494. 31687835 10.1259/bjr.20190494 PMC7066963

[43] Marchesini M, Ogoti Y, Fiorini E, Aktas Samur A, Nezi L, D’Anca M, et al. ILF2 is a regulator of RNA splicing and DNA damage response in 1q21-amplified multiple myeloma. Cancer Cell. 2017;32(1):88-100 e6. 10.1016/j.ccell.2017.05.011. 28669490 10.1016/j.ccell.2017.05.011 PMC5593798

[44] Eliseeva IA, Kim ER, Guryanov SG, Ovchinnikov LP, Lyabin DN. Y-box-binding protein 1 (YB-1) and its functions. Biochemistry (Mosc). 2011;76(13):1402–33. 10.1134/S0006297911130049. 22339596 10.1134/S0006297911130049

[45] Kuwano M, Shibata T, Watari K, Ono M. Oncogenic Y-box binding protein-1 as an effective therapeutic target in drug-resistant cancer. Cancer Sci. 2019;110(5):1536–43. 10.1111/cas.14006. 30903644 10.1111/cas.14006 PMC6500994

[46] Shibahara K, Uchiumi T, Fukuda T, Kura S, Tominaga Y, Maehara Y, et al. Targeted disruption of one allele of the Y-box binding protein-1 (YB-1) gene in mouse embryonic stem cells and increased sensitivity to cisplatin and mitomycin C. Cancer Sci. 2004;95(4):348–53. 10.1111/j.1349-7006.2004.tb03214.x. 15072594 10.1111/j.1349-7006.2004.tb03214.x PMC11159763

[47] Koike K, Uchiumi T, Ohga T, Toh S, Wada M, Kohno K, et al. Nuclear translocation of the Y-box binding protein by ultraviolet irradiation. FEBS Lett. 1997;417(3):390–4. 10.1016/s0014-5793(97)01296-9. 9409758 10.1016/s0014-5793(97)01296-9

[48] Zhao Y, Zhu M, Yu Y, Qiu L, Zhang Y, He L, et al. Brain REST/NRSF is not only a silent repressor but also an active protector. Mol Neurobiol. 2017;54(1):541–50. 10.1007/s12035-015-9658-4. 26742529 10.1007/s12035-015-9658-4

[49] Fuller GN, Su X, Price RE, Cohen ZR, Lang FF, Sawaya R, et al. Many human medulloblastoma tumors overexpress repressor element-1 silencing transcription (REST)/neuron-restrictive silencer factor, which can be functionally countered by REST-VP16. Mol Cancer Ther. 2005;4(3):343–9. 10.1158/1535-7163.MCT-04-0228. 15767543 10.1158/1535-7163.MCT-04-0228

[50] Abrajano JJ, Qureshi IA, Gokhan S, Molero AE, Zheng D, Bergman A, et al. Corepressor for element-1-silencing transcription factor preferentially mediates gene networks underlying neural stem cell fate decisions. Proc Natl Acad Sci U S A. 2010;107(38):16685–90. 10.1073/pnas.0906917107. 20823235 10.1073/pnas.0906917107 PMC2944745

[51] Coulson JM. Transcriptional regulation: cancer, neurons and the REST. Curr Biol. 2005;15(17):R665–8. 10.1016/j.cub.2005.08.032. 16139198 10.1016/j.cub.2005.08.032

[52] Su XJ, Shen BD, Wang K, Song QX, Yang X, Wu DS, et al. Roles of the neuron-restrictive silencer factor in the pathophysiological process of the central nervous system. Front Cell Dev Biol. 2022;10:834620. 10.3389/fcell.2022.834620. 35300407 10.3389/fcell.2022.834620 PMC8921553

[53] Pierce AM, Keating AK. Creating anatomically accurate and reproducible intracranial xenografts of human brain tumors. J Vis Exp. 2014;91:52017. 10.3791/52017. 10.3791/52017 PMC4694722 25285381

