# Supplementary material for: Prolactin-Releasing Hormone Receptor (PRLHR) enhances radiosensitivity and exacerbates DNA damage in glioblastoma post-irradiation by inhibiting Y-box-binding protein-1 (YBX1) nuclear translocation: a novel perspective on precision radiotherapy
Source: Mol Biomed. 2026 Jun 29;7:102. doi: 10.1186/s43556-026-00503-7 (PMC13310861; doi:10.1186/s43556-026-00503-7)
Supplement: Supplementary file 1 — Supplementary Material 1. [file 43556_2026_503_MOESM1_ESM.docx]

**Prolactin-Releasing Hormone Receptor (PRLHR) enhances radiosensitivity and exacerbates DNA damage in glioblastoma post-irradiation by inhibiting Y-Box-Binding Protein-1 (YBX1) nuclear translocation: A novel perspective on precision radiotherapy**

Yuning Qiu^1,2#^, Jing Zhang^3#^, Jingdian Liu^2#^, Zilong Wang^2^, Zeyu Ma^2^, Minkai Wang^2^, Qimeng Wang^4^, Xianzhi Liu^2*^, Dongming Yan^2*^ and Zhenyu Zhang^2*^

^1^Department of Surgery ICU, The First Affiliated Hospital of Zhengzhou University, Zhengzhou, Henan, China

^2^Department of Neurosurgery, The First Affiliated Hospital of Zhengzhou University, Zhengzhou, Henan, China

^3^Department of Pathology, Henan Provincial Chest Hospital, Zhengzhou, Henan, China

^4^Department of Pathology, The First Affiliated Hospital of Zhengzhou University, Zhengzhou, Henan, China

* Yuning Qiu, Jing Zhang, Jingdian Liu contributed equally to this work.

Corresponding authors:

Zhenyu Zhang, Department of Neurosurgery, The First Affiliated Hospital of Zhengzhou University, Jian she Dong Road 1, Zhengzhou, Henan province, China, 480082. Tel: +86-17839973727, E-mail: [fcczhangzy1@zzu.edu.cn](mailto:fcczhangzy1@zzu.edu.cn)

Dongming Yan, Department of Neurosurgery, The First Affiliated Hospital of Zhengzhou University, Jian she Dong Road 1, Zhengzhou, Henan province, China, 480082. Tel: +86-0371-67966157, E-mail: yandm@zzu.edu.cn

Xianzhi Liu, Department of Neurosurgery, The First Affiliated Hospital of Zhengzhou University, Jian she Dong Road 1, Zhengzhou, Henan province, China, 480082. Tel: +86-13803899299, E-mail: [fccliuxz@zzu.edu.cn](mailto:fccliuxz@zzu.edu.cn)

**Supplementary information**

**Supplementary Fig. S1**


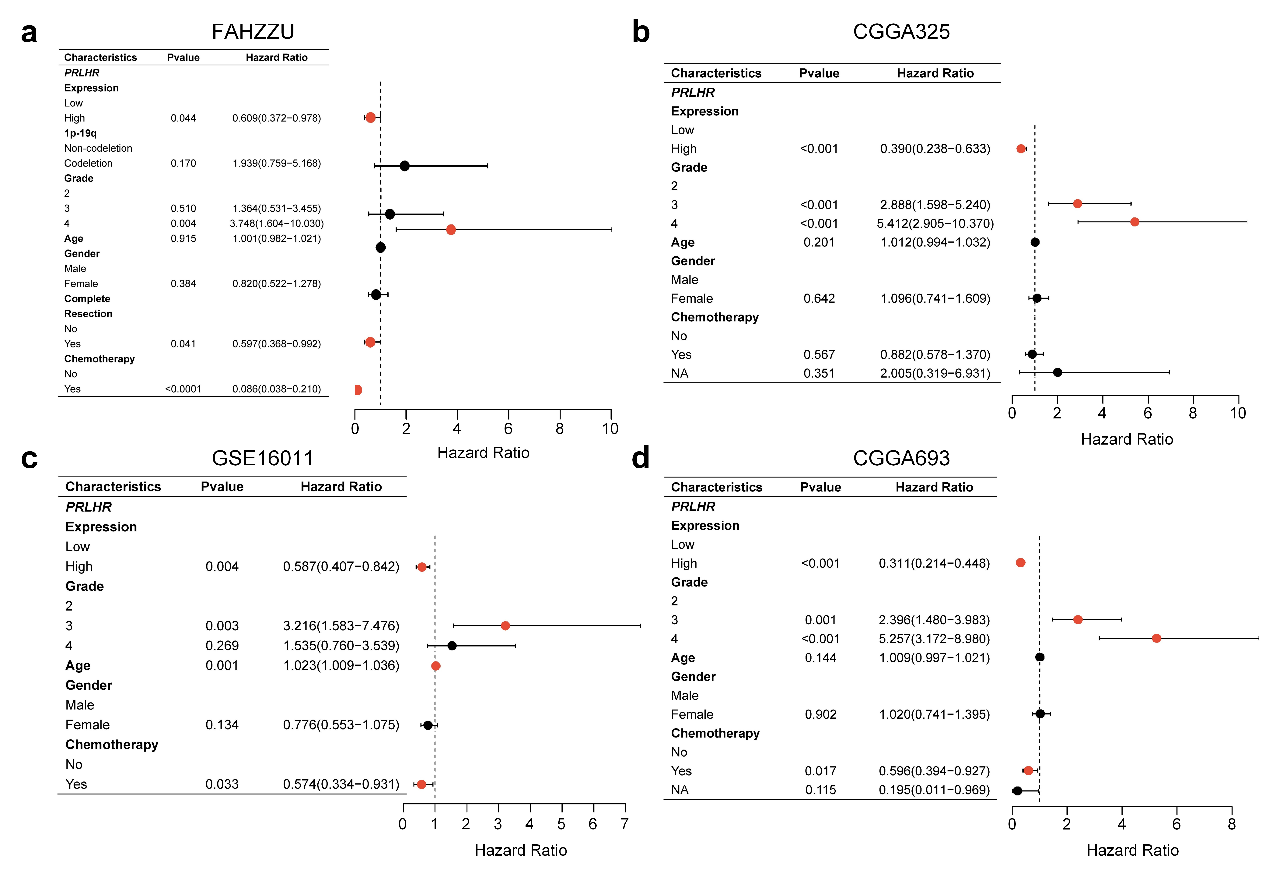


**Fig. S1 Cox proportional hazards regression model of clinical factors in glioma patients** **undergoing radiotherapy.**

(a-d) Cox proportional hazards regression models performed in the FAHZZU (a), CGGA325 (b), GSE16011 (c), and CGGA693 (d) datasets, incorporating age, sex,PRLHR expression level, WHO grade, resection extent, 1p/19q codeletion status, and chemotherapy treatment.

**Supplementary Fig. S2**


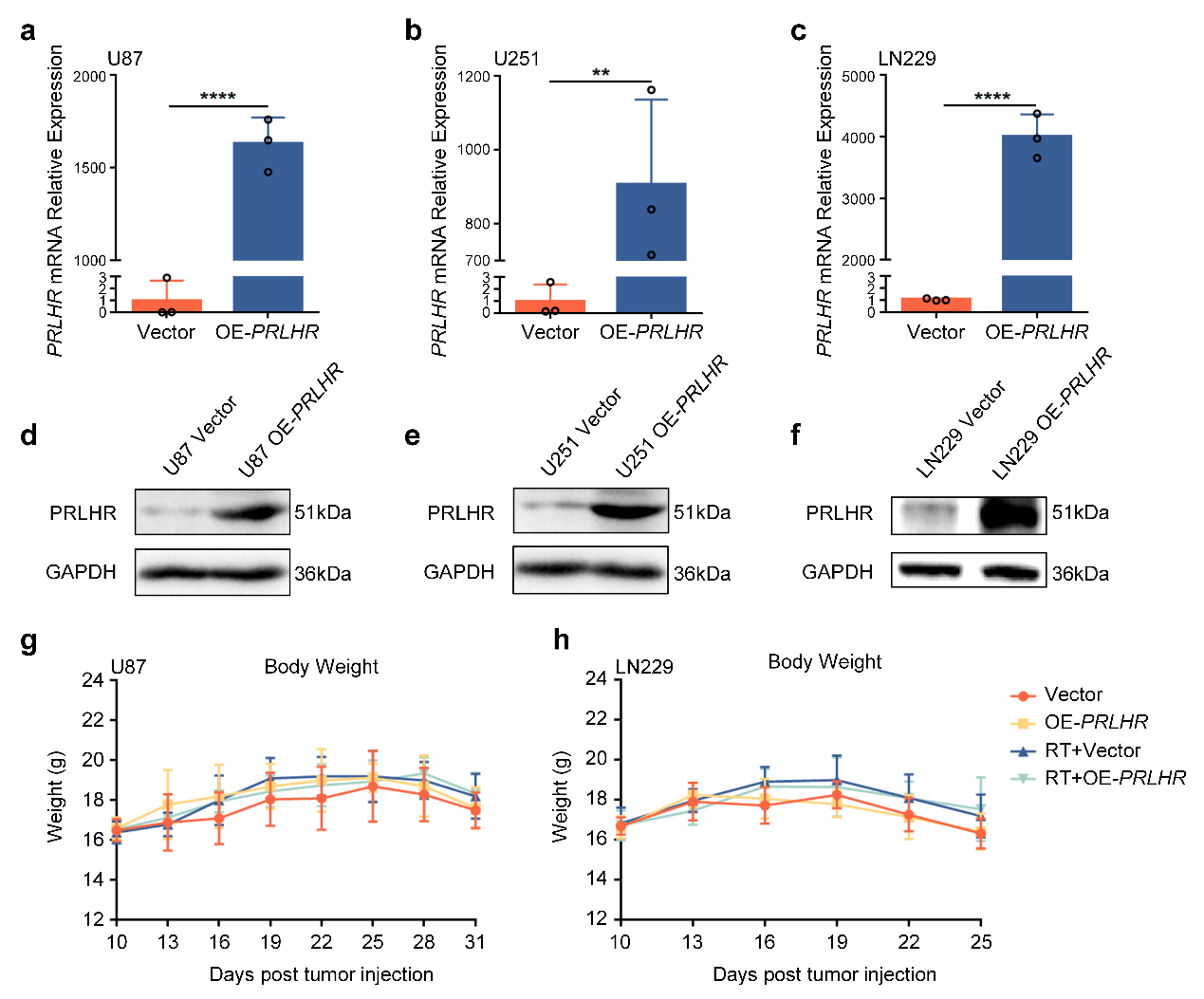


**Fig. S2 PRLHR overexpression enhances radiosensitivity in GBM cells**

(a-c) The mRNA expression levels of *PRLHR* in U87 (a), U251 (b) and LN229 (c) cells were detected by quantitative real-time polymerase chain reaction (qPCR)(n=3); (d-f) The protein expression levels of PRLHR in U87 (d), U251 (e) and LN229 (f) cells were determined by Western blot analysis. (g, h) Body weight changes in mice bearing orthotopic U87 (g) and LN229 (h) GBM intracranial tumor models (n=5).

**Supplementary Fig. S3**


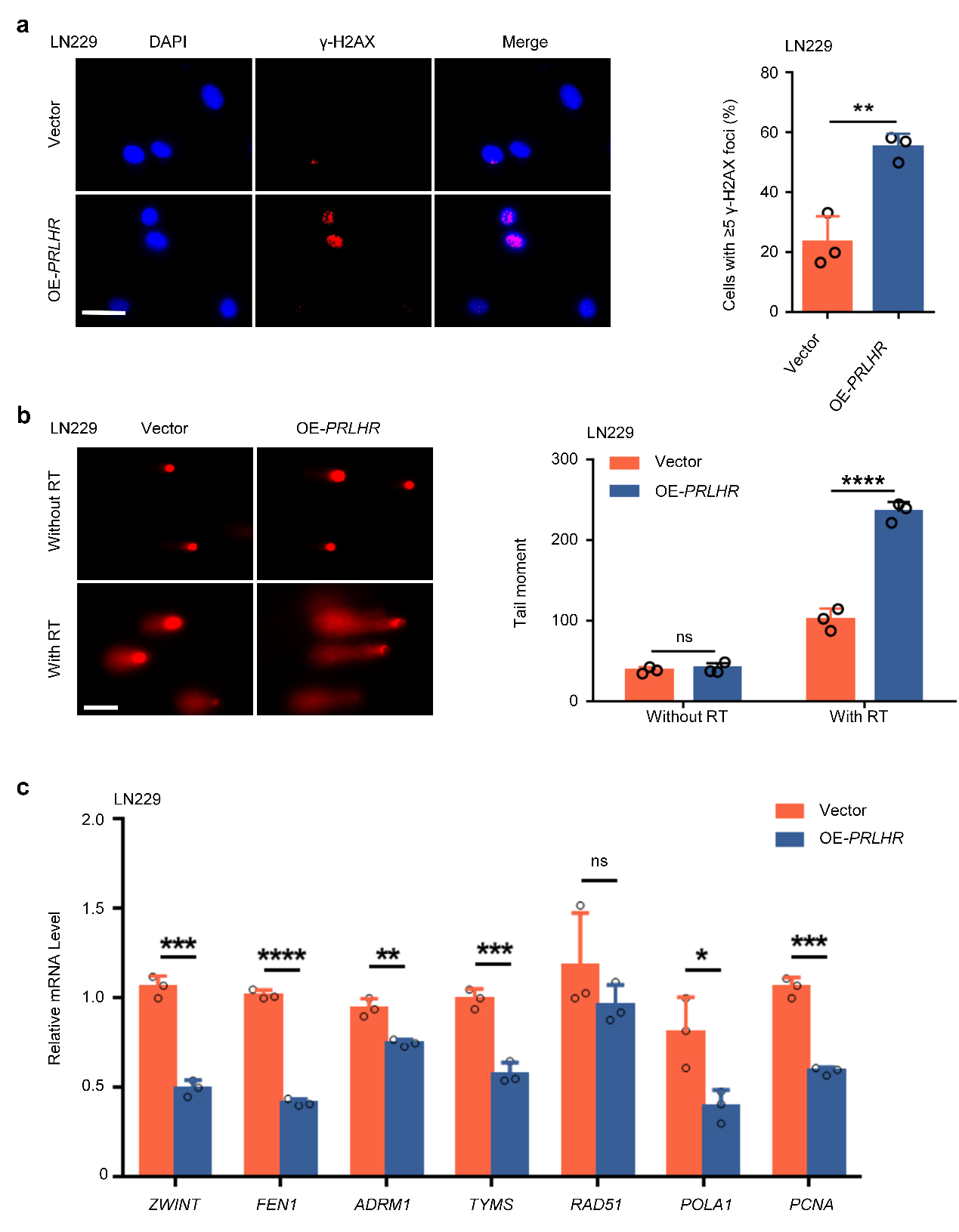


**Fig. S3 PRLHR inhibits DNA double-strand break repair in GBM cells following irradiation.**

(a) Detection of DNA DSBs assessed by γ-H2AX levels in LN229 cells at 24 hours post-irradiation (4 Gy) (n=3). Left panels: Representative immunofluorescence images of γ-H2AX foci; Right panels: Quantification of γ-H2AX foci per cell. Scale bars, 50 μm. (b) total DNA damage in irradiated (4 Gy, 24 h post-irradiation) and non-irradiated LN229 cells detected by comet assay(n=3). Left panels: Representative fluorescence images of comet assays; Right panels: Quantification of tail moment. Scale bars, 50 μm. (c) qPCR analysis of expression changes in DNA repair-related genes in *PRLHR*-overexpressing LN229 cells. *p< 0.05, **p< 0.01, ***p< 0.001, and ****p< 0.0001 by Student’s t-test.

**Supplementary Fig. S4**


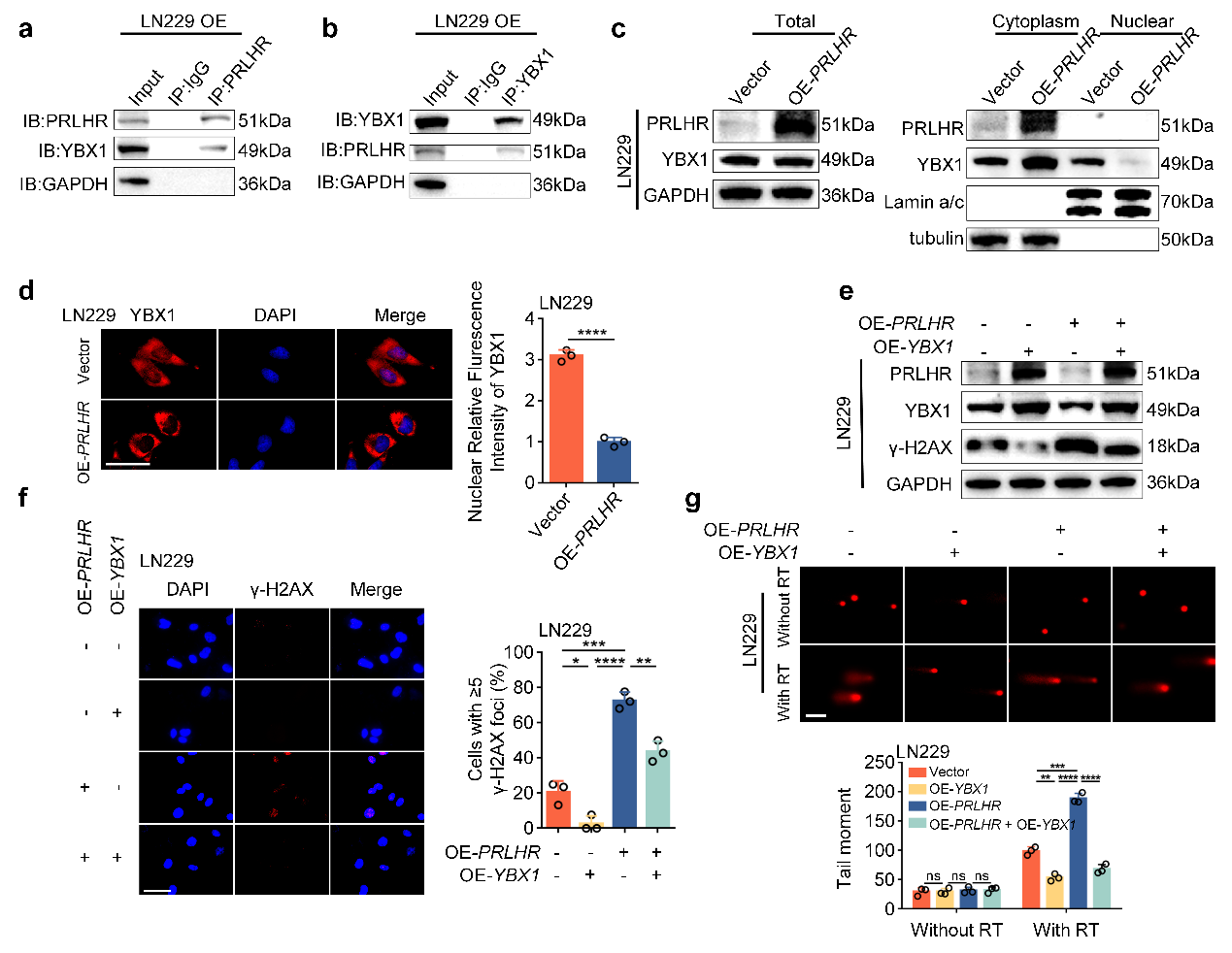


**Fig. S4 PRLHR binds to YBX1 and inhibits its nuclear translocation, thereby increasing DNA damage.** (a, b) Western blot analysis of PRLHR and YBX1 proteins using co-immunoprecipitation with anti-PRLHR (a) or anti-YBX1 (b) antibodies in LN229 OE cells. (c) Western blot detection of PRLHR and YBX1 expression levels in whole-cell lysates, cytoplasmic fractions, and nuclear fractions of LN229 cells. (d) Immunofluorescence analysis of the subcellular localization of YBX1 in LN229 cells. Left panels: Representative images of YBX1 subcellular localization(n=3). Scale bar, 50 μm. Right panels: Quantification of the relative nuclear fluorescence intensity of YBX1. (e) Western blot analysis of γ-H2AX expression levels in LN229 cells with *PRLHR* and/or *YBX1* overexpression following 4 Gy irradiation for 24 hours. (f) Immunofluorescence detection of γ-H2AX foci in *PRLHR* and/or *YBX1* overexpressing LN229 cells at 24 hours post-irradiation (4 Gy) (n=3). Left panels show representative γ-H2AX foci; Right panels show quantification data. Scale bars, 50 μm. (g) Comet assay was performed to detect total DNA damage in irradiated (4 Gy, 24 h post-irradiation) and non-irradiated LN229 cells with overexpression of *PRLHR* and/or *YBX1* (n=3). Upper panels show representative comet assay fluorescence images; Lower panels show quantification data. Scale bars, 50 μm. *p < 0.05, **p < 0.01, ***p < 0.001, and ****p < 0.0001.

**Supplementary Fig. S5**


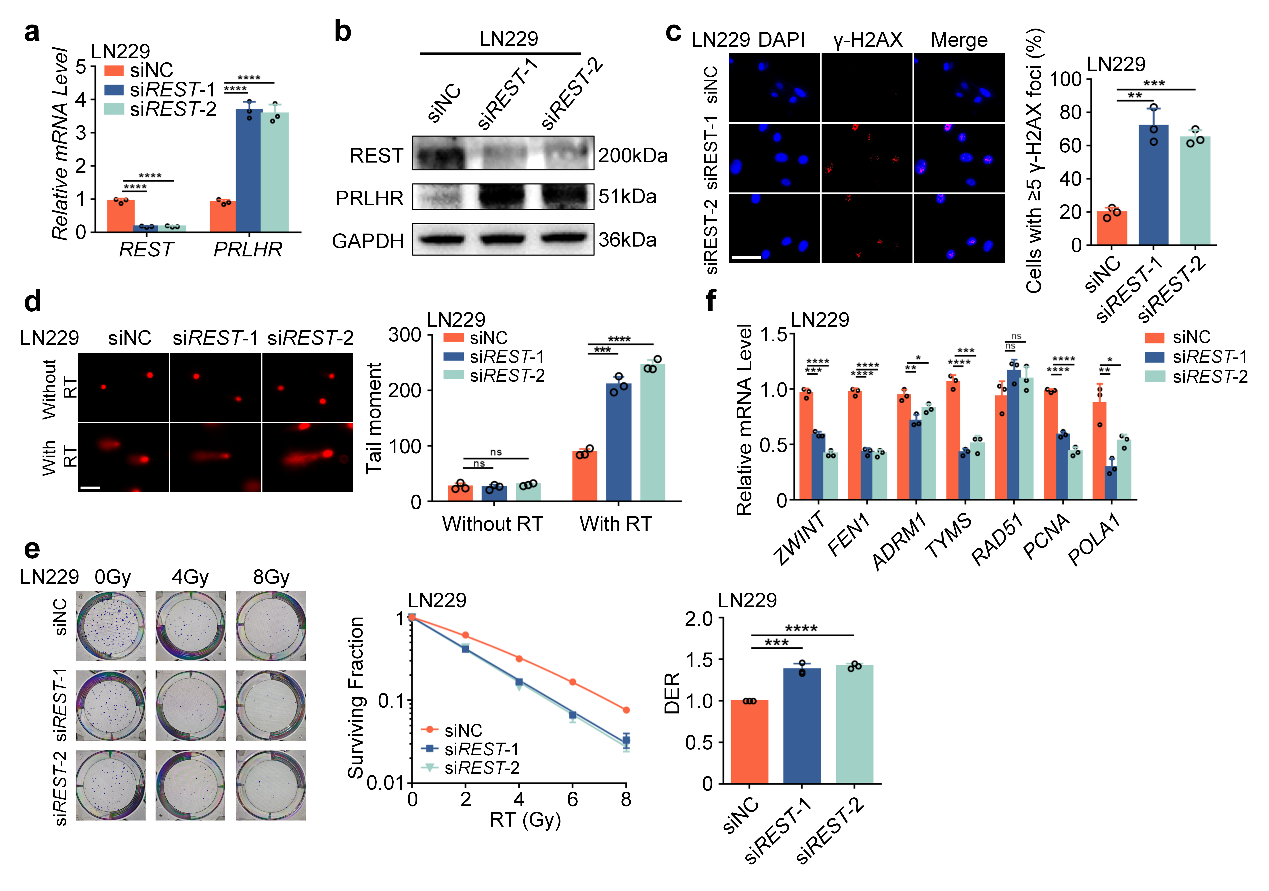


**Fig. S5 REST binds to the *PRLHR* promoter and negatively regulates PRLHR expression.** (a) qPCR analysis of *PRLHR* mRNA expression following *REST* knockdown in LN229 cells (n=3). (b) Western blot detection of PRLHR protein levels after *REST* knockdown in LN229 cells. (c) Immunofluorescence analysis of γ-H2AX foci in *REST*-knockdown LN229 cells at 24 hours post-irradiation (4 Gy) (n=3). Left panels: representative γ-H2AX foci; Right panels: quantitative data. Scale bars, 50 μm. (d) Comet assay was performed to detect total DNA damage in irradiated (4 Gy, 24 h post-irradiation) and non-irradiated *REST* knockdown LN229 cells (n=3). Left panels: representative comet assay images; Right panels: quantification data. Scale bars, 50 μm. (e) Colony formation assay analyzing cell survival and dose enhancement ratio (DER) in *REST*-knockdown LN229 cells (n=3). Left panels: representative colony images; Middle panels: survival fractions at different radiation doses, with curves fitted using the linear-quadratic (LQ) model; Right panels: Dose enhancement ratio (DER). (f) qPCR analysis of DNA repair-related gene expression changes in *REST*-knockdown LN229 cells (n=3). *p < 0.05, **p < 0.01, ***p < 0.001, and ****p < 0.0001 by Student’s t-test.

**Supplementary Fig. S6**


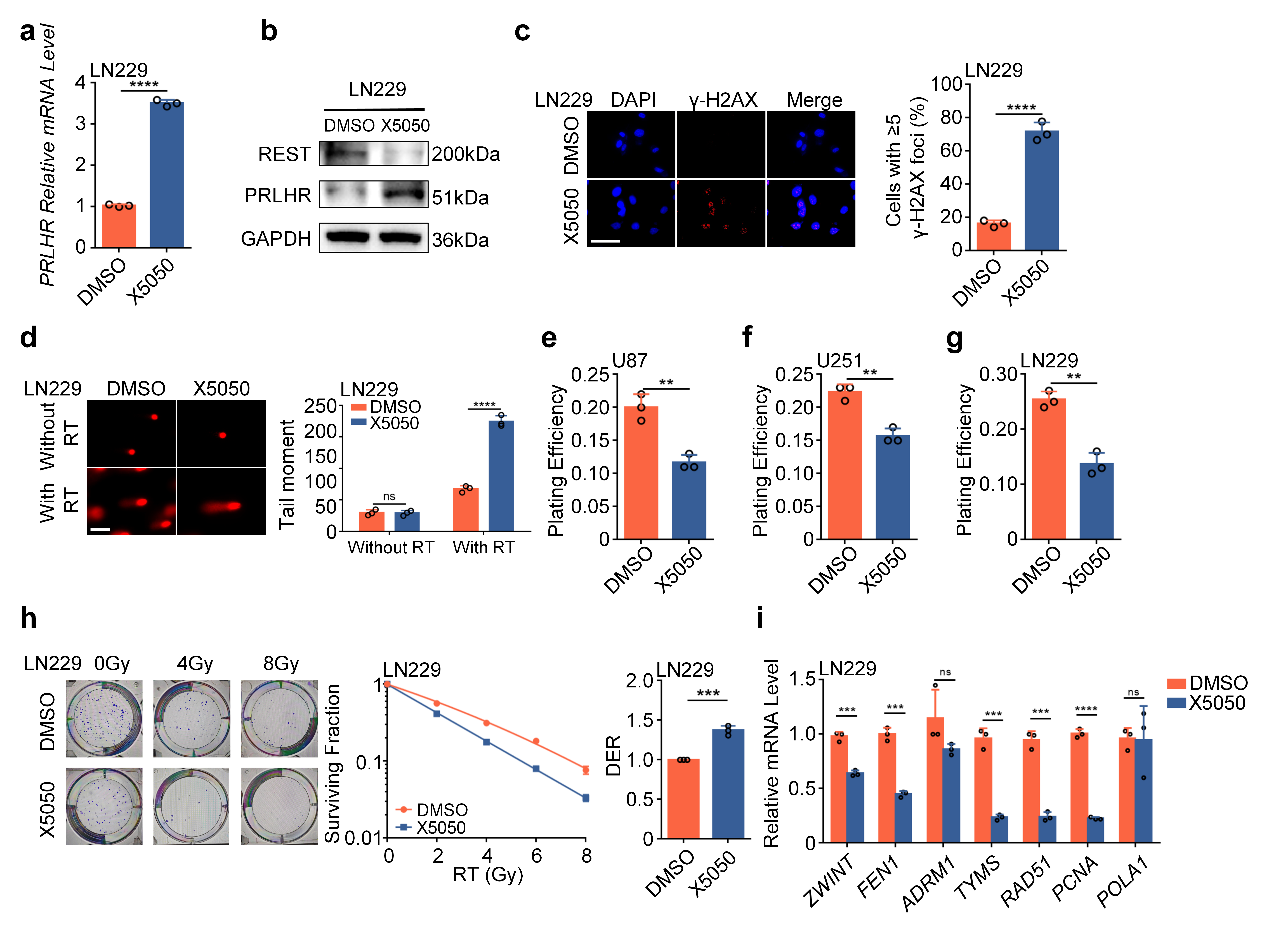


**Fig. S6 REST inhibitor X5050 enhances GBM radiosensitivity by increasing PRLHR expression.** (a) qPCR analysis of *PRLHR* mRNA expression levels in LN229 cells treated with X5050 (100 µM for 48 h) (n=3). (b) Western blot analysis of PRLHR protein expression levels in LN229 cells treated with X5050 (100 µM for 48 h). (c) Immunofluorescence was used to detect γ-H2AX foci in LN229 cells treated with X5050 (100 µM, 48 h) followed by irradiation (4 Gy) and incubation for 24 h (n=3). Left panels: Representative images of γ-H2AX foci. Right panels: Quantification data. Scale bars, 50 μm. (d) Comet assay was performed to detect total DNA damage in LN229 cells treated with X5050 (100 µM, 48 h) followed by irradiation (4 Gy, 24 h post-irradiation) or non-irradiation (n=3). Left panels: Representative comet assay fluorescence images. Right panels: Quantification data (tail moment). Scale bars, 50 μm. (e-g) Plating efficiency (PE) of U87 (e), U251 (f), and LN229 (g) cells treated with DMSO or X5050 (0 Gy). PE was calculated as the ratio of the number of colonies formed to the number of cells seeded. (h) Clonogenic survival assay for LN229 cells treated with X5050 (100 µM for 48 h) (n=3). Left panels: Representative images of colonies. Middle panels: Survival fractions at different radiation doses, with curves fitted using the linear-quadratic (LQ) model; Right panels: Dose enhancement ratio (DER). (i) qPCR analysis of DNA repair-related gene expression changes in LN229 cells treated with X5050 (100 µM for 48 h) (n=3). *p < 0.05, **p < 0.01, ***p < 0.001, ****p < 0.0001.

**Supplementary Fig. S7**


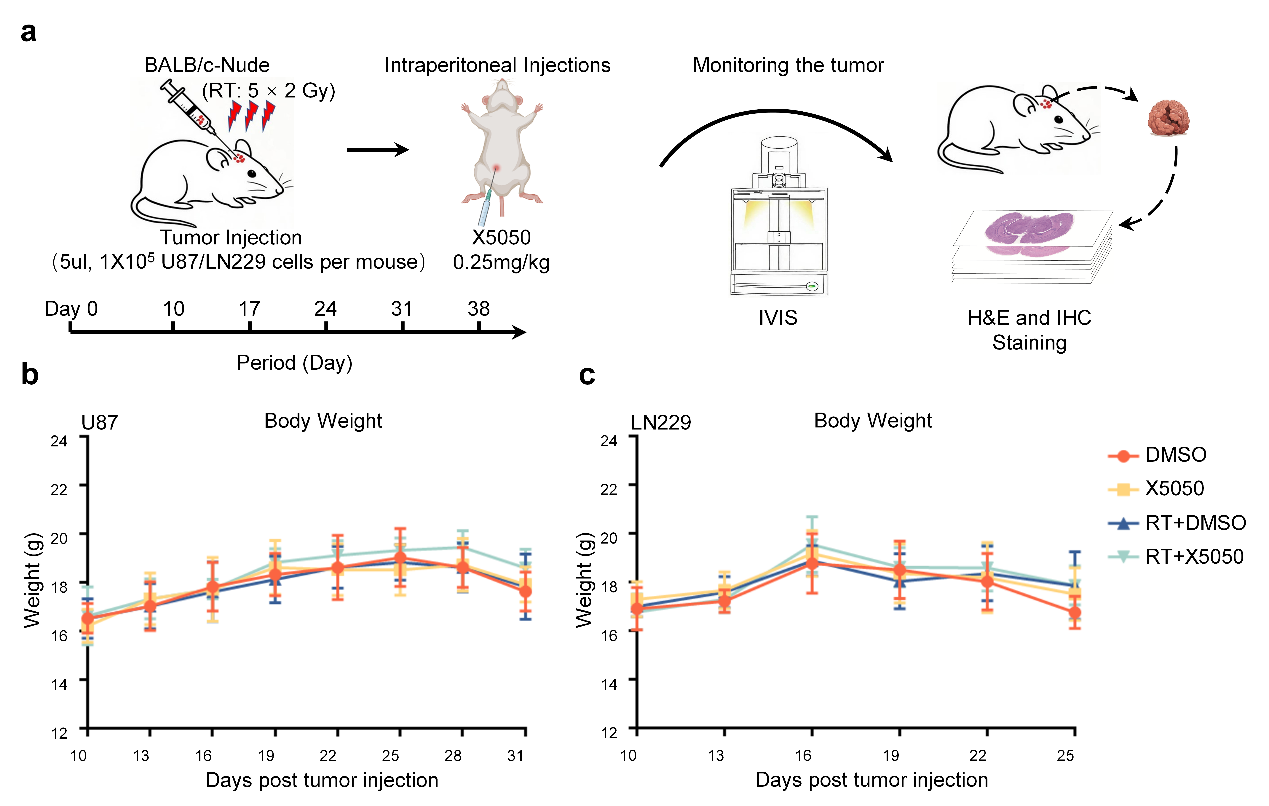


**Fig. S7** **REST inhibitor enhances the therapeutic efficacy of radiotherapy in GBM in vivo.**

(a) Schematic representation of the experimental timeline for the orthotopic GBM model in BALB/c nude mice. U87 or LN229 cells (1 × 10⁵) were implanted intracranially. 10 days post-implantation, mice were randomized into four groups: DMSO, X5050, RT + DMSO, and RT + X5050 (n = 5 per group). Mice were subjected to fractionated radiotherapy (5 × 2 Gy) and/or intraperitoneal (i.p.) administration of X5050 (0.25 mg/kg, twice weekly). Upon study termination, mice were euthanized, and tumor tissues were harvested for H&E and immunohistochemical (IHC) staining. (b, c) Time-dependent changes in body weight of mice bearing U87 (b) and LN229 (c) tumors treated with DMSO, X5050, RT+DMSO, or RT+X505

**Table S1 The clinical data of 144 glioma patients**

| Patients_ID | Age | Gender | Grade | 1p19q_codeletion | Resection | RT | CHT | Recurrence | PFS (months) | Status | OS (months) |
| --- | --- | --- | --- | --- | --- | --- | --- | --- | --- | --- | --- |
| Patient_1 | 39 | Male | 2 | Yes | No | Yes | Yes | Yes | 1 | dead | 10 |
| Patient_2 | 19 | Male | 4 | No | No | Yes | Yes | Yes | 1.3 | dead | 6.6 |
| Patient_3 | 48 | Male | 4 | No | Yes | Yes | Yes | Yes | 2.2 | dead | 7.8 |
| Patient_4 | 50 | Male | 4 | No | Yes | Yes | Yes | Yes | 3 | dead | 6.5 |
| Patient_5 | 49 | Male | 4 | No | Yes | Yes | Yes | Yes | 3 | dead | 7.8 |
| Patient_6 | 28 | Female | 4 | No | Yes | Yes | Yes | Yes | 3.9 | dead | 11.2 |
| Patient_7 | 78 | Female | 4 | No | Yes | Yes | Yes | Yes | 3.95 | dead | 8.95 |
| Patient_8 | 59 | Female | 4 | No | Yes | Yes | Yes | Yes | 5.3 | dead | 8.6 |
| Patient_9 | 27 | Male | 4 | No | Yes | Yes | Yes | Yes | 5.8 | dead | 11.1 |
| Patient_10 | 56 | Male | 4 | No | No | Yes | Yes | Yes | 6.6 | dead | 18 |
| Patient_11 | 44 | Male | 2 | No | No | Yes | Yes | Yes | 6.7 | alive | 50 |
| Patient_12 | 56 | Female | 4 | No | Yes | Yes | Yes | Yes | 7.1 | dead | 22.1 |
| Patient_13 | 59 | Male | 4 | No | No | Yes | Yes | Yes | 7.4 | dead | 14.72 |
| Patient_14 | 70 | Female | 4 | No | Yes | Yes | Yes | Yes | 7.6 | dead | 14.4 |
| Patient_15 | 57 | Male | 4 | No | Yes | Yes | Yes | Yes | 8.6 | dead | 10.9 |
| Patient_16 | 42 | Male | 4 | No | Yes | Yes | Yes | Yes | 9 | dead | 22.37 |
| Patient_17 | 65 | Male | 4 | No | Yes | Yes | Yes | Yes | 10.8 | dead | 30.55 |
| Patient_18 | 61 | Male | 4 | No | Yes | Yes | Yes | Yes | 10.9 | dead | 29.35 |
| Patient_19 | 50 | Female | 4 | No | Yes | Yes | Yes | Yes | 12 | dead | 21.93 |
| Patient_20 | 23 | Female | 4 | No | Yes | Yes | Yes | Yes | 12.3 | dead | 23.5 |
| Patient_21 | 44 | Female | 4 | No | Yes | Yes | Yes | Yes | 13.53 | dead | 17.53 |
| Patient_22 | 30 | Male | 4 | No | Yes | Yes | Yes | Yes | 14.6 | dead | 21.7 |
| Patient_23 | 67 | Male | 4 | No | Yes | Yes | Yes | Yes | 15.3 | dead | 23.21 |
| Patient_24 | 63 | Male | 4 | No | Yes | Yes | Yes | Yes | 15.8 | dead | 21.8 |
| Patient_25 | 60 | Male | 4 | No | Yes | Yes | Yes | Yes | 15.8 | dead | 21.8 |
| Patient_26 | 40 | Male | 4 | No | No | Yes | Yes | Yes | 16.8 | dead | 34.4 |
| Patient_27 | 55 | Female | 4 | No | Yes | Yes | Yes | Yes | 17.2 | dead | 17.2 |
| Patient_28 | 51 | Male | 4 | No | Yes | Yes | Yes | Yes | 18 | alive | 19.8 |
| Patient_29 | 71 | Male | 4 | No | Yes | Yes | Yes | Yes | 19 | dead | 22.65 |
| Patient_30 | 35 | Male | 3 | No | Yes | Yes | Yes | Yes | 19.26 | alive | 53.77 |
| Patient_31 | 56 | Male | 4 | No | Yes | Yes | Yes | Yes | 19.9 | dead | 19.9 |
| Patient_32 | 46 | Female | 4 | No | No | Yes | Yes | Yes | 21 | alive | 24.83 |
| Patient_33 | 58 | Female | 4 | No | Yes | Yes | Yes | Yes | 21.2 | dead | 28.2 |
| Patient_34 | 66 | Female | 4 | No | Yes | Yes | Yes | Yes | 21.8 | dead | 29.6 |
| Patient_35 | 61 | Male | 4 | No | Yes | Yes | Yes | Yes | 22.3 | alive | 53.27 |
| Patient_36 | 57 | Male | 4 | No | No | Yes | Yes | Yes | 27 | dead | 39.8 |
| Patient_37 | 62 | Male | 4 | No | Yes | Yes | Yes | Yes | 28.8 | alive | 33.2 |
| Patient_38 | 54 | Male | 4 | No | Yes | Yes | Yes | Yes | 29.5 | alive | 49.53 |
| Patient_39 | 72 | Female | 4 | No | Yes | Yes | Yes | Yes | 30.1 | dead | 32.1 |
| Patient_40 | 49 | Male | 4 | No | Yes | Yes | Yes | Yes | 30.3 | dead | 39.45 |
| Patient_41 | 48 | Female | 4 | No | Yes | Yes | Yes | Yes | 31.3 | dead | 37.8 |
| Patient_42 | 50 | Female | 4 | No | Yes | Yes | Yes | Yes | 32.5 | dead | 37.85 |
| Patient_43 | 65 | Female | 4 | No | Yes | Yes | Yes | Yes | 33.05 | dead | 39.15 |
| Patient_44 | 31 | Female | 4 | No | Yes | Yes | Yes | Yes | 33.35 | dead | 40.25 |
| Patient_45 | 45 | Male | 4 | No | No | Yes | Yes | Yes | 33.6 | dead | 40.15 |
| Patient_46 | 19 | Female | 4 | No | Yes | Yes | Yes | Yes | 34.6 | alive | 46.7 |
| Patient_47 | 47 | Male | 4 | No | Yes | Yes | Yes | Yes | 35.07 | alive | 43.4 |
| Patient_48 | 56 | Female | 4 | No | Yes | Yes | Yes | Yes | 38.5 | alive | 43.83 |
| Patient_49 | 46 | Female | 4 | No | Yes | Yes | Yes | Yes | 40.33 | alive | 52.73 |
| Patient_50 | 60 | Female | 4 | No | Yes | Yes | Yes | Yes | 42.7 | alive | 44 |
| Patient_51 | 50 | Female | 3 | Yes | No | Yes | Yes | Yes | 28.2 | dead | 33.1 |
| Patient_52 | 42 | Male | 2 | Yes | Yes | Yes | Yes | Yes | 31.3 | alive | 49.51 |
| Patient_53 | 30 | Female | 4 | No | Yes | Yes | Yes | Yes | 4 | dead | 8 |
| Patient_54 | 58 | Female | 4 | No | Yes | Yes | Yes | Yes | 5 | dead | 5 |
| Patient_55 | 18 | Female | 4 | No | Yes | Yes | Yes | Yes | 5.7 | dead | 7.4 |
| Patient_56 | 26 | Female | 4 | No | Yes | Yes | Yes | Yes | 6.2 | dead | 8 |
| Patient_57 | 59 | Male | 4 | No | No | Yes | Yes | Yes | 12 | dead | 18.2 |
| Patient_58 | 27 | Male | 3 | No | No | Yes | Yes | Yes | 16.1 | dead | 25.6 |
| Patient_59 | 55 | Female | 4 | No | Yes | Yes | Yes | Yes | 21.1 | dead | 38.4 |
| Patient_60 | 68 | Female | 4 | No | Yes | Yes | Yes | Yes | 24.43 | dead | 25.43 |
| Patient_61 | 64 | Female | 4 | No | Yes | Yes | Yes | Yes | 27.43 | alive | 32.77 |
| Patient_62 | 46 | Female | 3 | No | Yes | Yes | Yes | Yes | 28.1 | alive | 49.1 |
| Patient_63 | 46 | Male | 4 | No | Yes | Yes | Yes | Yes | 31.07 | alive | 47.04 |
| Patient_64 | 64 | Female | 4 | No | Yes | Yes | Yes | Yes | 32.57 | dead | 48.3 |
| Patient_65 | 46 | Male | 4 | No | No | Yes | Yes | Yes | 33 | dead | 48.9 |
| Patient_66 | 58 | Male | 4 | No | Yes | Yes | Yes | Yes | 10 | dead | 16.4 |
| Patient_67 | 35 | Male | 4 | No | Yes | Yes | Yes | Yes | 15.5 | dead | 19.9 |
| Patient_68 | 65 | Male | 4 | No | Yes | Yes | Yes | Yes | 16.73 | dead | 22.73 |
| Patient_69 | 63 | Female | 4 | No | Yes | Yes | Yes | Yes | 19 | dead | 19 |
| Patient_70 | 49 | Female | 4 | No | Yes | Yes | Yes | Yes | 20 | alive | 49.63 |
| Patient_71 | 62 | Male | 4 | No | Yes | Yes | Yes | Yes | 23.93 | dead | 43.47 |
| Patient_72 | 64 | Male | 3 | No | No | Yes | Yes | Yes | 31.2 | dead | 41.3 |
| Patient_73 | 33 | Female | 3 | No | Yes | Yes | Yes | Yes | 32.7 | dead | 39.1 |
| Patient_74 | 48 | Female | 2 | Yes | Yes | Yes | Yes | Yes | 34.6 | dead | 41.3 |
| Patient_75 | 39 | Male | 3 | No | No | Yes | Yes | Yes | 11.95 | alive | 37.8 |
| Patient_76 | 43 | Female | 4 | No | Yes | Yes | Yes | Yes | 15.5 | alive | 22.8 |
| Patient_77 | 32 | Male | 3 | No | Yes | Yes | Yes | Yes | 22 | alive | 30.7 |
| Patient_78 | 69 | Female | 4 | No | Yes | Yes | Yes | Yes | 32.4 | alive | 38.38 |
| Patient_79 | 44 | Male | 4 | No | No | Yes | Yes | Yes | 1.5 | dead | 8.3 |
| Patient_80 | 63 | Male | 4 | No | No | Yes | Yes | Yes | 1.8 | dead | 15.4 |
| Patient_81 | 46 | Male | 2 | No | Yes | Yes | Yes | Yes | 11.46 | alive | 41.93 |
| Patient_82 | 46 | Male | 4 | No | Yes | Yes | Yes | Yes | 20.1 | dead | 27.1 |
| Patient_83 | 74 | Female | 4 | No | Yes | Yes | Yes | Yes | 29.88 | alive | 40.4 |
| Patient_84 | 52 | Male | 2 | Yes | No | Yes | Yes | Yes | 15 | dead | 15 |
| Patient_85 | 54 | Male | 3 | Yes | No | Yes | Yes | Yes | 18 | alive | 31.1 |
| Patient_86 | 54 | Male | 4 | No | Yes | Yes | Yes | Yes | 13.5 | alive | 18.5 |
| Patient_87 | 56 | Female | 2 | Yes | Yes | Yes | Yes | Yes | 12.8 | alive | 30 |
| Patient_88 | 59 | Female | 4 | No | Yes | Yes | Yes | Yes | 31 | alive | 37 |
| Patient_89 | 69 | Male | 4 | No | Yes | Yes | Yes | Yes | 14.6 | alive | 32.1 |
| Patient_90 | 30 | Female | 4 | No | Yes | Yes | Yes | Yes | 33 | alive | 40 |
| Patient_91 | 70 | Male | 4 | No | Yes | Yes | Yes | Yes | 6.5 | dead | 6.5 |
| Patient_92 | 51 | Female | 4 | No | Yes | Yes | Yes | Yes | 6.8 | dead | 17.5 |
| Patient_93 | 61 | Female | 4 | No | Yes | Yes | Yes | Yes | 27 | alive | 31 |
| Patient_94 | 56 | Male | 4 | No | Yes | Yes | Yes | Yes | 6 | dead | 10 |
| Patient_95 | 62 | Female | 4 | No | Yes | Yes | Yes | Yes | 19 | dead | 21.7 |
| Patient_96 | 34 | Female | 4 | No | Yes | Yes | Yes | Yes | 22.7 | alive | 53 |
| Patient_97 | 49 | Male | 2 | Yes | No | Yes | Yes | Yes | 31.1 | alive | 40.1 |
| Patient_98 | 56 | Female | 4 | No | No | Yes | Yes | Yes | 11 | alive | 41.87 |
| Patient_99 | 51 | Male | 4 | No | Yes | Yes | Yes | Yes | 32.6 | dead | 41.3 |
| Patient_100 | 67 | Female | 4 | No | Yes | Yes | Yes | Yes | 3 | dead | 12.9 |
| Patient_101 | 56 | Female | 2 | No | No | Yes | Yes | Yes | 15.2 | alive | 50.26 |
| Patient_102 | 49 | Male | 3 | Yes | No | Yes | Yes | Yes | 30 | alive | 41.7 |
| Patient_103 | 47 | Male | 4 | No | Yes | Yes | Yes | Yes | 10.09 | dead | 23.3 |
| Patient_104 | 33 | Male | 2 | No | Yes | Yes | Yes | Yes | 16.9 | dead | 22.9 |
| Patient_105 | 46 | Male | 4 | No | Yes | Yes | No | Yes | 20.1 | dead | 28.2 |
| Patient_106 | 69 | Male | 4 | No | Yes | Yes | Yes | Yes | 8 | dead | 16 |
| Patient_107 | 41 | Male | 4 | No | No | Yes | Yes | Yes | 21 | dead | 30.6 |
| Patient_108 | 48 | Female | 4 | No | No | Yes | Yes | Yes | 35.13 | alive | 45 |
| Patient_109 | 57 | Female | 4 | No | No | Yes | Yes | Yes | 15.8 | dead | 22.6 |
| Patient_110 | 77 | Male | 3 | Yes | Yes | Yes | Yes | Yes | 18.5 | alive | 30.5 |
| Patient_111 | 28 | Male | 2 | No | Yes | Yes | Yes | Yes | 23 | alive | 33.7 |
| Patient_112 | 53 | Male | 3 | Yes | No | Yes | Yes | Yes | 28 | dead | 37 |
| Patient_113 | 50 | Female | 4 | No | Yes | Yes | Yes | Yes | 33.3 | alive | 42.35 |
| Patient_114 | 27 | Female | 4 | No | No | Yes | Yes | Yes | 2.1 | dead | 2.1 |
| Patient_115 | 52 | Female | 4 | No | Yes | Yes | Yes | Yes | 18.1 | alive | 43.4 |
| Patient_116 | 64 | Female | 2 | Yes | Yes | Yes | Yes | Yes | 25.3 | alive | 44.27 |
| Patient_117 | 42 | Female | 3 | Yes | Yes | Yes | Yes | Yes | 13.5 | dead | 20.5 |
| Patient_118 | 41 | Male | 3 | No | No | Yes | Yes | Yes | 11 | dead | 19.5 |
| Patient_119 | 44 | Male | 4 | No | No | Yes | Yes | Yes | 16 | dead | 26 |
| Patient_120 | 53 | Male | 4 | No | Yes | Yes | Yes | Yes | 18.5 | dead | 24.67 |
| Patient_121 | 53 | Male | 2 | No | Yes | Yes | Yes | Yes | 36.53 | alive | 41.1 |
| Patient_122 | 57 | Female | 4 | No | No | Yes | Yes | Yes | 31.8 | dead | 38.6 |
| Patient_123 | 39 | Male | 2 | No | Yes | Yes | Yes | Yes | 43.7 | alive | 48.7 |
| Patient_124 | 34 | Male | 2 | No | Yes | Yes | Yes | Yes | 14.2 | dead | 21.7 |
| Patient_125 | 64 | Male | 4 | No | No | Yes | Yes | Yes | 3 | dead | 10.2 |
| Patient_126 | 57 | Male | 4 | No | Yes | Yes | Yes | Yes | 1.2 | dead | 22.67 |
| Patient_127 | 51 | Female | 2 | Yes | No | Yes | Yes | Yes | 10 | dead | 24.2 |
| Patient_128 | 55 | Male | 3 | Yes | Yes | Yes | Yes | Yes | 13 | alive | 25.17 |
| Patient_129 | 37 | Female | 3 | No | Yes | Yes | Yes | Yes | 24.8 | alive | 51.37 |
| Patient_130 | 25 | Male | 2 | No | Yes | Yes | Yes | Yes | 33.6 | alive | 40.2 |
| Patient_131 | 61 | Male | 4 | No | Yes | Yes | Yes | Yes | 26.2 | dead | 32.6 |
| Patient_132 | 34 | Male | 2 | No | No | Yes | Yes | Yes | 2.1 | dead | 5 |
| Patient_133 | 46 | Male | 2 | No | Yes | Yes | Yes | Yes | 43.3667 | alive | 47.87 |
| Patient_134 | 38 | Male | 2 | Yes | No | Yes | Yes | Yes | 27 | alive | 29.4 |
| Patient_135 | 66 | Male | 2 | Yes | No | Yes | Yes | Yes | 30.9 | dead | 36.6 |
| Patient_136 | 29 | Male | 2 | No | Yes | Yes | No | Yes | 22.25 | dead | 22.25 |
| Patient_137 | 47 | Female | 2 | No | Yes | Yes | Yes | Yes | 23 | alive | 39.1 |
| Patient_138 | 51 | Male | 2 | Yes | No | Yes | Yes | Yes | 23 | alive | 33.5 |
| Patient_139 | 49 | Female | 2 | Yes | Yes | Yes | Yes | Yes | 39.5 | alive | 46.65 |
| Patient_140 | 25 | Male | 2 | No | Yes | Yes | Yes | Yes | 37.17 | alive | 46 |
| Patient_141 | 29 | Male | 2 | Yes | Yes | Yes | Yes | Yes | 31.5 | dead | 38.1 |
| Patient_142 | 39 | Female | 3 | Yes | No | Yes | Yes | Yes | 33.5 | dead | 43 |
| Patient_143 | 34 | Female | 3 | Yes | No | Yes | Yes | Yes | 6 | dead | 15.15 |
| Patient_144 | 42 | Male | 2 | Yes | Yes | Yes | Yes | Yes | 29 | alive | 39.1 |

**Table S2 Sequences of primers for RT-qPCR**

| *PRLHR* | Forward primer | CTGCACAACGTGACGAACTT |
| --- | --- | --- |
|  | Reward primer | GCGTGAACACCGACACATAG |
| *ZWINT* | Forward primer | GCATCTTGGAACCTGTAGGC |
|  | Reward primer | GCCTTCAGCTCTTTCCATTG |
| *FEN1* | Forward primer | ACATGGACTGCCTCACCTTC |
|  | Reward primer | CCCAATACCCCGGATACTCT |
| *ADRM1* | Forward primer | GACGGACGACTCGCTTATTC |
|  | Reward primer | TCCTGGTCTGTCTTGGGTTC |
| *TYMS* | Forward primer | TCTGGAAGGGTGTTTTGGAG |
|  | Reward primer | CCTCCACTGGAAGCCATAAA |
| *RAD51* | Forward primer | TTTGGAGAATTCCGAACTGG |
|  | Reward primer | CATCACTGCCAGAGAGACCA |
| *POLA1* | Forward primer | GCCAGCAGAGGAAGTGAAAC |
|  | Reward primer | CCCTTTTACCAATGGGAGGT |
| *PCNA* | Forward primer | GGCGTGAACCTCACCAGTAT |
|  | Reward primer | TCTCGGCATATACGTGCAAA |
| *GAPDH* | Forward primer | GGAGTCCACTGGCGTCTTCA |
|  | Reward primer | GTCATGAGTCCTTCCACGATACC |

**Table S3 Potential PRLHR-interacting proteins in U87 OE cells identified using mass spectrometry**

| Protein accession | Protein names | Gene names | MW [kDa] | Protein score | Sequence coverage (%) | Unique Peptides | Peptides | PSMs | Abundances |
| --- | --- | --- | --- | --- | --- | --- | --- | --- | --- |
| Q9BQE3 | Tubulin alpha-1C chain | TUBA1C | 49.9 | 1333 | 53 | 6 | 23 | 39 | 7.36e+08 |
| Q9UNF1 | Melanoma-associated antigen D2 | MAGED2 | 64.9 | 552 | 28 | 16 | 16 | 19 | 2.08e+08 |
| P67809 | Y-box-binding protein 1 | YBX1 | 35.9 | 506 | 54 | 13 | 13 | 15 | 1.22e+08 |
| O43493 | Trans-Golgi network integral membrane protein 2 | TGOLN2 | 45.9 | 484 | 29 | 10 | 10 | 12 | 4.16e+07 |
| Q9NVP1 | ATP-dependent RNA helicase DDX18 | DDX18 | 75.4 | 355 | 14 | 8 | 8 | 9 | 4.65e+07 |
| P19338 | Nucleolin | NCL | 76.6 | 307 | 11 | 7 | 7 | 8 | 3.90e+07 |
| O60271 | C-Jun-amino-terminal kinase-interacting protein 4 | SPAG9 | 146.1 | 254 | 11 | 10 | 10 | 10 | 2.69e+07 |
| P09651 | Heterogeneous nuclear ribonucleoprotein A1 | HNRNPA1 | 38.7 | 239 | 14 | 3 | 4 | 6 | 1.54e+07 |
| P19474 | E3 ubiquitin-protein ligase TRIM21 | TRIM21 | 54.1 | 223 | 15 | 7 | 7 | 8 | 9.49e+07 |
| O00425 | Insulin-like growth factor 2 mRNA-binding protein 3 | IGF2BP3 | 63.7 | 190 | 12 | 5 | 6 | 6 | 1.96e+07 |
| P17858 | ATP-dependent 6-phosphofructokinase, liver type | PFKL | 85 | 172 | 6 | 1 | 3 | 4 | 2.92e+06 |
| O75367 | Core histone macro-H2A.1 | MACROH2A1 | 39.2 | 167 | 17 | 4 | 4 | 4 | 1.49e+07 |
| Q96CV9 | Optineurin | OPTN | 65.9 | 147 | 6 | 3 | 3 | 3 | 1.17e+07 |
| Q8NB90 | ATPase family gene 2 protein homolog A | AFG2A | 97.8 | 141 | 5 | 4 | 4 | 5 | 1.66e+07 |
| P78318 | Immunoglobulin-binding protein 1 | IGBP1 | 39.2 | 140 | 16 | 4 | 4 | 4 | 1.79e+07 |
| Q6ZMR3 | L-lactate dehydrogenase A-like 6A | LDHAL6A | 36.5 | 140 | 6 | 1 | 3 | 3 | 2.60e+06 |
| P38159 | RNA-binding motif protein, X chromosome | RBMX | 42.3 | 133 | 18 | 6 | 6 | 7 | 2.71e+07 |
| P29692 | Elongation factor 1-delta | EEF1D | 31.1 | 127 | 20 | 4 | 4 | 4 | 1.07e+07 |
| O43707 | Alpha-actinin-4 | ACTN4 | 104.8 | 127 | 5 | 3 | 3 | 3 | 5.62e+06 |
| P51991 | Heterogeneous nuclear ribonucleoprotein A3 | HNRNPA3 | 39.6 | 126 | 8 | 1 | 2 | 2 | 1.27e+06 |
| Q06210 | Glutamine--fructose-6-phosphate aminotransferase [isomerizing] 1 | GFPT1 | 78.8 | 123 | 9 | 3 | 5 | 5 | 7.97e+06 |
| O15027 | Protein transport protein Sec16A | SEC16A | 251.7 | 120 | 2 | 3 | 3 | 4 | 1.61e+07 |
| P01024 | Complement C3 | C3 | 187 | 116 | 2 | 2 | 2 | 3 | 3.38e+07 |
| Q13310 | Polyadenylate-binding protein 4 | PABPC4 | 70.7 | 111 | 8 | 2 | 5 | 5 | 5.64e+06 |
| O75477 | Erlin-1 | ERLIN1 | 39.1 | 104 | 9 | 1 | 3 | 3 | 6.46e+06 |
| Q9NVI7 | ATPase family AAA domain-containing protein 3A | ATAD3A | 71.3 | 101 | 4 | 1 | 3 | 3 | 8.09e+06 |
| P06396 | Gelsolin | GSN | 85.6 | 100 | 6 | 5 | 5 | 5 | 1.25e+07 |
| Q7Z2W4 | Zinc finger CCCH-type antiviral protein 1 | ZC3HAV1 | 101.4 | 99 | 3 | 2 | 2 | 2 | 6.93e+06 |
| Q99733 | Nucleosome assembly protein 1-like 4 | NAP1L4 | 42.8 | 91 | 5 | 2 | 2 | 2 | 6.72e+06 |
| Q9P035 | Very-long-chain (3R)-3-hydroxyacyl-CoA dehydratase 3 | HACD3 | 43.1 | 90 | 6 | 3 | 3 | 3 | 1.00e+07 |
| P83881 | Large ribosomal subunit protein eL42 | RPL36A | 12.4 | 90 | 25 | 1 | 3 | 3 | 1.26e+07 |
| O43795 | Unconventional myosin-Ib | MYO1B | 131.9 | 89 | 4 | 4 | 4 | 4 | 1.97e+07 |
| Q96F07 | Cytoplasmic FMR1-interacting protein 2 | CYFIP2 | 148.3 | 87 | 3 | 3 | 3 | 3 | 1.58e+07 |
| P27169 | Serum paraoxonase/arylesterase 1 | PON1 | 39.7 | 83 | 3 | 1 | 1 | 2 | 1.42e+06 |
| Q92901 | Ribosomal protein uL3-like | RPL3L | 46.3 | 82 | 7 | 1 | 4 | 4 | 4.20e+06 |
| Q969V3 | BOS complex subunit NCLN | NCLN | 62.9 | 78 | 5 | 2 | 2 | 3 | 6.06e+06 |
| P26599 | Polypyrimidine tract-binding protein 1 | PTBP1 | 59.6 | 78 | 2 | 1 | 1 | 1 | 4.92e+06 |
| O75146 | Huntingtin-interacting protein 1-related protein | HIP1R | 119.3 | 78 | 1 | 1 | 1 | 1 | 1.61e+06 |
| P46087 | 28S rRNA (cytosine(4447)-C(5))-methyltransferase | NOP2 | 89.2 | 76 | 2 | 1 | 1 | 1 | 1.53e+06 |
| Q16666 | Gamma-interferon-inducible protein 16 | IFI16 | 88.2 | 73 | 7 | 4 | 4 | 4 | 1.11e+07 |
| Q7Z417 | FMR1-interacting protein NUFIP2 | NUFIP2 | 76.1 | 73 | 4 | 2 | 2 | 2 | 6.93e+06 |
| P40938 | Replication factor C subunit 3 | RFC3 | 40.5 | 71 | 4 | 1 | 1 | 1 | 1.07e+06 |
| Q5T9A4 | ATPase family AAA domain-containing protein 3B | ATAD3B | 72.5 | 70 | 5 | 1 | 3 | 3 | 4.26e+06 |
| P39019 | Small ribosomal subunit protein eS19 | RPS19 | 16.1 | 70 | 14 | 2 | 2 | 2 | 6.59e+06 |
| Q9BQ39 | ATP-dependent RNA helicase DDX50 | DDX50 | 82.5 | 69 | 3 | 1 | 2 | 2 | 1.08e+07 |
| Q99575 | Ribonucleases P/MRP protein subunit POP1 | POP1 | 114.6 | 68 | 4 | 3 | 3 | 3 | 3.48e+06 |
| P46977 | Dolichyl-diphosphooligosaccharide--protein glycosyltransferase subunit STT3A | STT3A | 80.5 | 67 | 2 | 1 | 1 | 1 | 1.66e+06 |
| Q16643 | Drebrin | DBN1 | 71.4 | 63 | 2 | 1 | 1 | 1 | 2.58e+06 |
| O96005 | Putative lipid scramblase CLPTM1 | CLPTM1 | 76 | 61 | 3 | 2 | 2 | 2 | 1.13e+07 |
| Q8TC12 | Retinol dehydrogenase 11 | RDH11 | 35.4 | 61 | 6 | 2 | 2 | 2 | 1.67e+06 |
| Q8NCA5 | Protein FAM98A | FAM98A | 55.2 | 60 | 2 | 1 | 1 | 1 |  |
| Q9Y5A9 | YTH domain-containing family protein 2 | YTHDF2 | 62.3 | 59 | 2 | 1 | 1 | 1 | 7.16e+05 |
| Q14202 | Zinc finger MYM-type protein 3 | ZMYM3 | 152.3 | 58 | 1 | 1 | 1 | 1 | 4.10e+06 |
| Q9H0A0 | RNA cytidine acetyltransferase | NAT10 | 115.7 | 57 | 2 | 2 | 2 | 2 | 7.18e+06 |
| P25398 | Small ribosomal subunit protein eS12 | RPS12 | 14.5 | 57 | 8 | 1 | 1 | 1 | 3.56e+06 |
| Q13501 | Sequestosome-1 | SQSTM1 | 47.7 | 57 | 6 | 1 | 1 | 1 | 2.45e+06 |
| Q8TCT9 | Minor histocompatibility antigen H13 | HM13 | 41.5 | 53 | 6 | 2 | 2 | 2 | 9.77e+06 |
| O95816 | BAG family molecular chaperone regulator 2 | BAG2 | 23.8 | 53 | 4 | 1 | 1 | 1 | 6.79e+06 |
| P51153 | Ras-related protein Rab-13 | RAB13 | 22.8 | 52 | 6 | 1 | 1 | 1 |  |
| P59190 | Ras-related protein Rab-15 | RAB15 | 24.4 | 51 | 5 | 1 | 1 | 1 | 7.58e+06 |
| Q01105 | Protein SET | SET | 33.5 | 51 | 3 | 1 | 1 | 1 | 7.66e+06 |
| Q9NYL9 | Tropomodulin-3 | TMOD3 | 39.6 | 51 | 3 | 1 | 1 | 1 | 2.29e+06 |
| P52926 | High mobility group protein HMGI-C | HMGA2 | 11.8 | 50 | 21 | 1 | 1 | 1 | 3.36e+06 |
| Q92621 | Nuclear pore complex protein Nup205 | NUP205 | 227.8 | 48 | 1 | 2 | 2 | 2 | 4.93e+06 |
| Q969M3 | Protein YIPF5 | YIPF5 | 28 | 48 | 5 | 1 | 1 | 1 | 2.72e+06 |
| P10620 | Microsomal glutathione S-transferase 1 | MGST1 | 17.6 | 46 | 8 | 1 | 1 | 1 |  |
| Q9BUJ2 | Heterogeneous nuclear ribonucleoprotein U-like protein 1 | HNRNPUL1 | 95.7 | 46 | 1 | 1 | 1 | 1 | 9.50e+05 |
| Q8TF72 | Protein Shroom3 | SHROOM3 | 216.7 | 45 | 0 | 1 | 1 | 2 |  |
| P16615 | Sarcoplasmic/endoplasmic reticulum calcium ATPase 2 | ATP2A2 | 114.7 | 44 | 2 | 2 | 2 | 2 |  |
| Q15773 | Myeloid leukemia factor 2 | MLF2 | 28.1 | 44 | 4 | 1 | 1 | 1 | 7.20e+05 |
| Q9H223 | EH domain-containing protein 4 | EHD4 | 61.1 | 43 | 5 | 1 | 2 | 2 | 1.23e+07 |
| P00846 | ATP synthase subunit a | MT-ATP6 | 24.8 | 42 | 4 | 1 | 1 | 1 | 3.48e+06 |
| Q27J81 | Inverted formin-2 | INF2 | 135.5 | 42 | 1 | 1 | 1 | 1 | 1.18e+06 |
| P62910 | Large ribosomal subunit protein eL32 | RPL32 | 15.9 | 41 | 15 | 2 | 2 | 2 | 2.66e+07 |
| Q96PK6 | RNA-binding protein 14 | RBM14 | 69.4 | 41 | 4 | 2 | 2 | 2 | 5.73e+06 |
| Q15166 | Serum paraoxonase/lactonase 3 | PON3 | 39.6 | 41 | 2 | 1 | 1 | 1 | 4.53e+06 |
| P62854 | Small ribosomal subunit protein eS26 | RPS26 | 13 | 41 | 13 | 1 | 1 | 1 | 9.34e+06 |
| Q8TDN6 | Ribosome biogenesis protein BRX1 homolog | BRIX1 | 41.4 | 41 | 3 | 1 | 1 | 1 | 3.41e+06 |
| P35241 | Radixin | RDX | 68.5 | 41 | 2 | 1 | 1 | 1 | 4.64e+06 |
| P47914 | Large ribosomal subunit protein eL29 | RPL29 | 17.7 | 39 | 9 | 1 | 1 | 1 | 8.14e+06 |
| Q9Y305 | Acyl-coenzyme A thioesterase 9, mitochondrial | ACOT9 | 49.9 | 39 | 3 | 1 | 1 | 1 | 1.54e+06 |
| Q9Y3U8 | Large ribosomal subunit protein eL36 | RPL36 | 12.2 | 39 | 9 | 1 | 1 | 1 | 3.00e+06 |
| P61009 | Signal peptidase complex subunit 3 | SPCS3 | 20.3 | 39 | 5 | 1 | 1 | 1 |  |
| P01599 | Immunoglobulin kappa variable 1-17 | IGKV1-17 | 12.8 | 38 | 13 | 1 | 1 | 1 |  |
| P25786 | Proteasome subunit alpha type-1 | PSMA1 | 29.5 | 37 | 3 | 1 | 1 | 1 | 3.42e+06 |
| O00429 | Dynamin-1-like protein | DNM1L | 81.8 | 37 | 2 | 1 | 1 | 1 | 2.48e+06 |
| Q12789 | General transcription factor 3C polypeptide 1 | GTF3C1 | 238.7 | 36 | 1 | 2 | 2 | 2 | 3.56e+06 |
| O75821 | Eukaryotic translation initiation factor 3 subunit G | EIF3G | 35.6 | 36 | 3 | 1 | 1 | 1 | 3.51e+06 |
| Q8N5M9 | Protein jagunal homolog 1 | JAGN1 | 21.1 | 36 | 7 | 1 | 1 | 1 |  |
| Q8TB61 | Adenosine 3'-phospho 5'-phosphosulfate transporter 1 | SLC35B2 | 47.5 | 36 | 2 | 1 | 1 | 1 |  |
| Q9Y5R4 | MTRF1L release factor glutamine methyltransferase | HEMK1 | 38.2 | 36 | 2 | 1 | 1 | 1 | 8.93e+06 |
| P62937 | Peptidyl-prolyl cis-trans isomerase A | PPIA | 18 | 36 | 7 | 1 | 1 | 1 |  |
| P16401 | Histone H1.5 | H1-5 | 22.6 | 35 | 10 | 1 | 2 | 2 | 4.73e+06 |
| O60716 | Catenin delta-1 | CTNND1 | 108.1 | 35 | 1 | 1 | 1 | 1 | 2.11e+06 |
| P51571 | Translocon-associated protein subunit delta | SSR4 | 19 | 35 | 6 | 1 | 1 | 1 |  |
| P49792 | E3 SUMO-protein ligase RanBP2 | RANBP2 | 358 | 35 | 0 | 1 | 1 | 1 |  |
| O75683 | Surfeit locus protein 6 | SURF6 | 41.4 | 35 | 2 | 1 | 1 | 1 | 4.44e+06 |
| Q13162 | Peroxiredoxin-4 | PRDX4 | 30.5 | 35 | 3 | 1 | 1 | 1 | 3.50e+06 |
| Q8IY81 | pre-rRNA 2'-O-ribose RNA methyltransferase FTSJ3 | FTSJ3 | 96.5 | 35 | 1 | 1 | 1 | 1 | 2.40e+06 |
| P50281 | Matrix metalloproteinase-14 | MMP14 | 65.9 | 34 | 2 | 1 | 1 | 1 |  |
| P62714 | Serine/threonine-protein phosphatase 2A catalytic subunit beta isoform | PPP2CB | 35.6 | 34 | 3 | 1 | 1 | 1 | 2.30e+06 |
| Q86TG7 | Retrotransposon-derived protein PEG10 | PEG10 | 80.1 | 33 | 1 | 1 | 1 | 1 | 3.63e+06 |
| P22626 | Heterogeneous nuclear ribonucleoproteins A2/B1 | HNRNPA2B1 | 37.4 | 33 | 4 | 1 | 1 | 1 | 3.83e+06 |
| Q9BQG0 | Myb-binding protein 1A | MYBBP1A | 148.8 | 32 | 1 | 1 | 1 | 1 | 2.67e+06 |
| Q9UQE7 | Structural maintenance of chromosomes protein 3 | SMC3 | 141.5 | 32 | 1 | 1 | 1 | 1 |  |
| A0A0B4J1U7 | Immunoglobulin heavy variable 6-1 | IGHV6-1 | 13.5 | 32 | 6 | 1 | 1 | 1 | 2.85e+08 |
| Q8TCJ2 | Dolichyl-diphosphooligosaccharide--protein glycosyltransferase subunit STT3B | STT3B | 93.6 | 31 | 1 | 1 | 1 | 1 | 3.90e+06 |
| O43237 | Cytoplasmic dynein 1 light intermediate chain 2 | DYNC1LI2 | 54.1 | 31 | 2 | 1 | 1 | 1 | 2.71e+06 |
| Q15582 | Transforming growth factor-beta-induced protein ig-h3 | TGFBI | 74.6 | 31 | 2 | 1 | 1 | 1 | 1.94e+06 |
| Q8TAM2 | Tetratricopeptide repeat protein 8 | TTC8 | 61.5 | 31 | 1 | 1 | 1 | 1 | 3.50e+06 |
| Q8IX12 | Cell division cycle and apoptosis regulator protein 1 | CCAR1 | 132.7 | 31 | 1 | 1 | 1 | 1 | 3.48e+06 |
| Q9NYJ8 | TGF-beta-activated kinase 1 and MAP3K7-binding protein 2 | TAB2 | 76.4 | 31 | 1 | 1 | 1 | 1 | 5.23e+06 |
| P17252 | Protein kinase C alpha type | PRKCA | 76.7 | 31 | 1 | 1 | 1 | 1 | 2.17e+06 |
| Q5VZE5 | N-alpha-acetyltransferase 35, NatC auxiliary subunit | NAA35 | 83.6 | 30 | 1 | 1 | 1 | 1 | 4.94e+06 |
| Q9NZ01 | Very-long-chain enoyl-CoA reductase | TECR | 36 | 30 | 3 | 1 | 1 | 1 | 5.87e+06 |
| Q8WZ64 | Arf-GAP with Rho-GAP domain, ANK repeat and PH domain-containing protein 2 | ARAP2 | 193.3 | 30 | 0 | 1 | 1 | 1 | 7.44e+07 |
| Q14739 | Delta(14)-sterol reductase LBR | LBR | 70.7 | 30 | 1 | 1 | 1 | 1 | 3.89e+06 |
| O60518 | Ran-binding protein 6 | RANBP6 | 124.6 | 30 | 1 | 1 | 1 | 1 | 2.38e+06 |
| Q6Y1H2 | Very-long-chain (3R)-3-hydroxyacyl-CoA dehydratase 2 | HACD2 | 28.4 | 30 | 4 | 1 | 1 | 1 | 2.43e+06 |
| O95478 | Ribosome biogenesis protein NSA2 homolog | NSA2 | 30 | 30 | 4 | 1 | 1 | 1 | 3.33e+06 |
| O15269 | Serine palmitoyltransferase 1 | SPTLC1 | 52.7 | 29 | 4 | 1 | 1 | 1 |  |
| Q9UBX7 | Kallikrein-11 | KLK11 | 31 | 29 | 2 | 1 | 1 | 1 | 7.71e+07 |
| Q08J23 | RNA cytosine C(5)-methyltransferase NSUN2 | NSUN2 | 86.4 | 29 | 1 | 1 | 1 | 1 | 1.59e+07 |
| Q9Y679 | Lipid droplet-regulating VLDL assembly factor AUP1 | AUP1 | 45.8 | 29 | 2 | 1 | 1 | 1 | 3.66e+06 |
| A6NNS2 | Dehydrogenase/reductase SDR family member 7C | DHRS7C | 34.9 | 29 | 5 | 1 | 1 | 1 |  |
| P17612 | cAMP-dependent protein kinase catalytic subunit alpha | PRKACA | 40.6 | 28 | 2 | 1 | 1 | 1 | 5.56e+06 |
| O75037 | Kinesin-like protein KIF21B | KIF21B | 182.5 | 28 | 0 | 1 | 1 | 1 |  |
| O94925 | Glutaminase kidney isoform, mitochondrial | GLS | 73.4 | 28 | 3 | 1 | 1 | 1 |  |
| P62333 | 26S proteasome regulatory subunit 10B | PSMC6 | 44.1 | 28 | 2 | 1 | 1 | 1 | 3.94e+06 |
| Q9NZB2 | Constitutive coactivator of PPAR-gamma-like protein 1 | FAM120A | 121.8 | 27 | 2 | 1 | 1 | 1 | 3.12e+06 |
| Q8WVY7 | Ubiquitin-like domain-containing CTD phosphatase 1 | UBLCP1 | 36.8 | 27 | 3 | 1 | 1 | 1 | 3.22e+06 |
| Q9Y2R4 | Probable ATP-dependent RNA helicase DDX52 | DDX52 | 67.4 | 27 | 1 | 1 | 1 | 1 | 1.21e+06 |
| Q9Y6V0 | Protein piccolo | PCLO | 560.4 | 27 | 0 | 1 | 1 | 1 | 2.32e+06 |
| Q8NBJ5 | Procollagen galactosyltransferase 1 | COLGALT1 | 71.6 | 27 | 1 | 1 | 1 | 1 |  |
| P27824 | Calnexin | CANX | 67.5 | 27 | 1 | 1 | 1 | 1 | 8.58e+06 |
| Q13045 | Protein flightless-1 homolog | FLII | 144.7 | 27 | 1 | 1 | 1 | 1 |  |
| Q12965 | Unconventional myosin-Ie | MYO1E | 127 | 27 | 2 | 1 | 1 | 1 | 2.60e+06 |
| Q99943 | 1-acyl-sn-glycerol-3-phosphate acyltransferase alpha | AGPAT1 | 31.7 | 27 | 4 | 1 | 1 | 1 | 2.38e+08 |
| Q9H6A9 | Pecanex-like protein 3 | PCNX3 | 221.9 | 26 | 0 | 1 | 1 | 1 | 1.09e+07 |
| Q12830 | Nucleosome-remodeling factor subunit BPTF | BPTF | 338.1 | 26 | 0 | 1 | 1 | 1 | 6.81e+06 |
| Q86TM6 | E3 ubiquitin-protein ligase synoviolin | SYVN1 | 67.6 | 26 | 1 | 1 | 1 | 1 | 1.27e+06 |
| P46782 | Small ribosomal subunit protein uS7 | RPS5 | 22.9 | 26 | 8 | 1 | 1 | 1 |  |
| Q14894 | Ketimine reductase mu-crystallin | CRYM | 33.8 | 26 | 3 | 1 | 1 | 1 | 4.68e+07 |
| Q15029 | 116 kDa U5 small nuclear ribonucleoprotein component | EFTUD2 | 109.4 | 25 | 1 | 1 | 1 | 1 | 2.47e+06 |
| P62841 | Small ribosomal subunit protein uS19 | RPS15 | 17 | 25 | 8 | 1 | 1 | 1 | 3.77e+06 |
| O14579 | Coatomer subunit epsilon | COPE | 34.5 | 25 | 7 | 1 | 1 | 1 |  |
| Q9Y230 | RuvB-like 2 | RUVBL2 | 51.1 | 25 | 3 | 1 | 1 | 1 |  |
| Q96EY7 | Small ribosomal subunit protein mS39 | PTCD3 | 78.5 | 25 | 1 | 1 | 1 | 1 | 1.91e+06 |
| Q5I0X7 | Tetratricopeptide repeat protein 32 | TTC32 | 17.3 | 25 | 4 | 1 | 1 | 1 | 1.80e+07 |
| Q05655 | Protein kinase C delta type | PRKCD | 77.5 | 24 | 2 | 1 | 1 | 1 | 3.19e+06 |
| A0A075B6I1 | Immunoglobulin lambda variable 4-60 | IGLV4-60 | 13 | 24 | 8 | 1 | 1 | 1 |  |
| Q14137 | Ribosome biogenesis protein BOP1 | BOP1 | 83.6 | 24 | 1 | 1 | 1 | 1 | 5.79e+06 |
| Q9BUP0 | EF-hand domain-containing protein D1 | EFHD1 | 26.9 | 23 | 5 | 1 | 1 | 1 | 6.23e+06 |
| P52292 | Importin subunit alpha-1 | KPNA2 | 57.8 | 23 | 3 | 1 | 1 | 1 |  |
| Q9BSJ2 | Gamma-tubulin complex component 2 | TUBGCP2 | 102.5 | 23 | 1 | 1 | 1 | 1 |  |
| O95486 | Protein transport protein Sec24A | SEC24A | 119.7 | 23 | 2 | 1 | 1 | 1 |  |
| O95747 | Serine/threonine-protein kinase OSR1 | OXSR1 | 58 | 21 | 2 | 1 | 1 | 1 | 4.30e+06 |
| Q9Y6K0 | Choline/ethanolaminephosphotransferase 1 | CEPT1 | 46.5 | 20 | 3 | 1 | 1 | 1 | 3.56e+06 |
